# Establishment, Validation, and Initial Application of a Sensitive LC-MS/MS Assay for Quantification of the Naturally Occurring Isomers Itaconate, Mesaconate, and Citraconate

**DOI:** 10.3390/metabo11050270

**Published:** 2021-04-26

**Authors:** Moritz Winterhoff, Fangfang Chen, Nishika Sahini, Thomas Ebensen, Maike Kuhn, Volkhard Kaever, Heike Bähre, Frank Pessler

**Affiliations:** 1 TWINCORE Centre for Experimental and Clinical Infection Research, 30625 Hannover, Germany; moritz.winterhoff@twincore.de (M.W.); Fangfang.Chen@twincore.de (F.C.); sahininishika@gmail.com (N.S.); maike.kuhn@t-online.de (M.K.); 2 Helmholtz Centre for Infection Research (HZI), 38124 Braunschweig, Germany; thomas.ebensen@helmholtz-hzi.de; 3 Research Core Unit Metabolomics, Hannover Medical School, 30625 Hannover, Germany; kaever.volkhard@mh-hannover.de (V.K.); baehre.heike@mh-hannover.de (H.B.); 4 Centre for Individualised Infection Medicine, 30625 Hannover, Germany

**Keywords:** biomarker, CAD, *cis*-aconitate decarboxylase, citraconate, Irg1, itaconate, Krebs cycle, mass spectrometry, mesaconate, metabolism

## Abstract

Itaconate is derived from the tricarboxylic acid (TCA) cycle intermediate *cis*-aconitate and links innate immunity and metabolism. Its synthesis is altered in inflammation-related disorders and it therefore has potential as clinical biomarker. Mesaconate and citraconate are naturally occurring isomers of itaconate that have been linked to metabolic disorders, but their functional relationships with itaconate are unknown. We aimed to establish a sensitive high performance liquid chromatography-tandem mass spectrometry (HPLC-MS/MS) assay for the quantification of itaconate, mesaconate, citraconate, the pro-drug 4-octyl-itaconate, and selected TCA intermediates. The assay was validated for itaconate, mesaconate, and citraconate for intra- and interday precision and accuracy, extended stability, recovery, freeze/thaw cycles, and carry-over. The lower limit of quantification was 0.098 µM for itaconate and mesaconate and 0.049 µM for citraconate in 50 µL samples. In spike-in experiments, itaconate remained stable in human plasma and whole blood for 24 and 8 h, respectively, whereas spiked-in citraconate and mesaconate concentrations changed during incubation. The type of anticoagulant in blood collection tubes affected measured levels of selected TCA intermediates. Human plasma may contain citraconate (0.4–0.6 µM, depending on the donor), but not itaconate or mesaconate, and lipopolysaccharide stimulation of whole blood induced only itaconate. Concentrations of the three isomers differed greatly among mouse organs: Itaconate and citraconate were most abundant in lymph nodes, mesaconate in kidneys, and only citraconate occurred in brain. This assay should prove useful to quantify itaconate isomers in biomarker and pharmacokinetic studies, while providing internal controls for their effects on metabolism by allowing quantification of TCA intermediates.

## 1. Introduction

The unsaturated dicarboxylic acid itaconic acid is a highly inducible byproduct of the tricarboxylic acid (TCA) cycle that is derived from *cis-*aconitate by *cis-*aconitate decarboxylase (ACOD1, also known as CAD, or immune responsive gene 1, Irg1) [1,2]. It has been in the limelight of research in the life and biomedical sciences ever since its discovery as a crucial link between immunity and metabolism in 2013 [1]. A variety of immunomodulatory and cytoprotective properties have been identified in addition to its previously postulated antibacterial effects [3,4,5]. Consequently, therapeutic efficacy of exogenously applied itaconate, or its methylated or octylated variants, has been demonstrated in preclinical models of infections [6,7], sterile inflammation [8], and ischemic reperfusion injury [9,10,11]. In addition, altered concentrations of itaconate in biological fluids, cells, or tissue have been found in animal diseases [12,13], a mouse model of arthritis [14], and in a variety of human diseases [13,15,16,17]. Human loss-of-function mutations in the enzyme catalyzing itaconate synthesis, ACOD1, are extremely rare, suggesting that physiological itaconate synthesis is important at the population level [2]. Table S1 in Supplementary Materials provides a summary of currently available biomarker studies featuring itaconate concentrations in humans or model animals. These studies were not based on rigorously validated measurement assays, and comparability across studies is limited due to the use of different methods and platforms. Nonetheless, the available evidence has clearly shown the potential of itaconate as a therapeutic intervention and potential biomarker for a variety of inflammation-related diseases.

**Figure 1:**
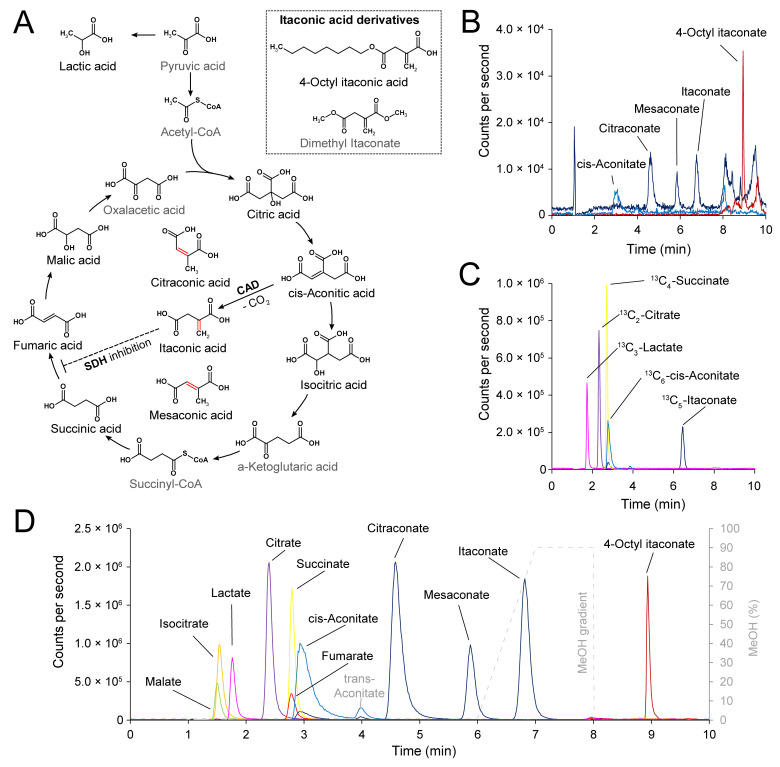
Detection of itaconate and isomers by high performance liquid chromatography-tandem mass spectrometry (HPLC-MS/MS). (**A**)—Chemical structures of tricarboxylic acid (TCA) cycle intermediates, itaconic acid isomers and derivatives. Compounds that can be quantified by the HPLC-MS/MS assay are highlighted in black. (**B**)—HPLC-MS/MS ion chromatogram of *cis-*aconitate, itaconic acid and isomers, and 4-octyl itaconate at the lower limit of quantification (LLOQ) (245 fmol on column for *cis-*aconitate and citraconate; 490 fmol on column for itaconate and mesaconate and 100 fmol on column for 4-octyl-itaconate). (**C**)—HPLC-MS/MS ion chromatograms of the employed internal standards. (**D**)—HPLC-MS/MS ion chromatograms of all analytes included in the method are highlighted in black. 4-octyl-itaconate eluted after applying the methanol gradient (dashed line in grey); *trans*-aconitate (grey) could be identified but was not quantified in this assay.

A currently underappreciated observation is that there are two naturally occurring isomers of itaconate, i.e., citraconate and mesaconate, which differ from it only by the location of the double bond (Figure 1A) and could potentially coexist with itaconate in human and animal biosamples. Both isomers have been detected in human biofluids, and altered concentrations can be linked to rare inborn errors of metabolism in humans and, in the case of citraconate, also to more common disorders. For instance, elevated citraconate concentrations have been found in urine from patients with ketosis due to methylmalonic acidemia, and it was suggested that it was derived via omega-oxidation of tiglyl-CoA during the ketotic state [18]. In addition, increased citraconate concentrations have been reported in colorectal cancer [19], type II diabetes [20], in sera from pregnant women with raised polychlorinated biphenyl levels [21], and in blood from patients with cirrhosis from viral hepatitis (compared to patients with primary biliary cholangitis) [22]. Raised mesaconate concentrations can be found in patients with isovaleric acidemia, where it is postulated to be derived from methylsuccinic acid, which is a catabolite of isovaleric acid [23]. Increased urine mesaconate concentrations were recently described in patients with congenital squalene synthase deficiency, likely as one of the catabolites of the squalene precursor farnesol, which accumulates in this disorder [24]. Tables S2 and S3 summarize biomarker studies of mesaconate and citraconate in humans and animal models.

In light of the data summarized above, the close structural similarities among itaconate, mesaconate, and citraconate raise the following questions: (1) To what extent is their synthesis or catabolism temporally or spatially interrelated? (2) Is there direct biological or chemical interconversion under certain circumstances? (3) Could artefactual interisomeric conversion in a detection assay lead to misidentification or inaccurate measurements? (4) Do the isomers share any disease associations? (5) Could they be developed into clinically usable biomarkers? (6) Can mesaconate and citraconate affect the immunomodulatory or antimicrobial functions of itaconate, or do they have similar effects of their own?

In order to address these questions and to advance the translational development of itaconate, mesaconate, and citraconate for diagnostic or therapeutic purposes, it is important to have a detection assay that can accurately discriminate among them and has the robustness required for studies geared towards clinical applications. In addition, this assay should provide robust quantification of lactate and TCA intermediates that are known to be regulated by itaconate, notably succinate, which accumulates due to itaconate-mediated inhibition of succinate dehydrogenase (SDH) [10,25]. This far, a variety of techniques, namely liquid chromatography-ultraviolet detection (LC-UV), nuclear magnetic resonance (NMR), gas chromatography-mass spectrometry (GC-MS) and liquid chromatography-mass spectrometry (LC-MS), have been used to quantify itaconate, mesaconate, or citraconate in individual assays (also see Tables S1–S3 for a summary of literature on itaconate isomer quantification methods in diseases and disease models). Each of these methods has its own strengths and weaknesses. LC-UV measurements may be affordable and broadly available but are not very sensitive and may be prone to interference in more complex matrices. This technique is suitable for the quantification of millimolar concentrations of itaconate in enzymatic in vitro assays [2,26] or in medium supernatants of, e.g., *Aspergillus terreus* strains that are used for industrial production of itaconate [27].

Higher resolution techniques such as NMR and MS are needed when these small organic acids are to be quantified reliably in the submillimolar range. While sample preparation for NMR may be simple and exceptionally nondestructive, this method lacks sensitivity and selectivity when compared to MS. Concerning relative itaconate quantification, NMR was, e.g., successfully used for untargeted analyses of organ extracts from *M. tuberculosis* and *S. typhimurium* infected mice [28,29].

Over the last decade, more sensitive analyses of itaconate levels in various biosamples have relied on GC-MS/MS methods, which require extensive sample preparation and derivatization of the analytes. These assays most commonly used methoxyamine-hydrochloride (MOX) and *N*-tert-butyldimethylsilyl-*N*-methyltrifluoroacetamide (MTBSTFA) or *N*-methyl-*N*-(trimethylsilyl)-trifluoroacetamide (MSTFA) derivatization under basic conditions in the presence of pyridine and temperatures ranging from 40 to 70 °C [1,9,10,16,17,25,30]. An important consideration is that methods requiring derivatization may lead to artefactual generation of itaconate and its isomers due to conversion of other molecules present in the sample matrix. Indeed, in our own preliminary studies for a GC-MS/MS based assay, we found that using pentafluorobenzyl bromide (PFB-Br [31]) in the presence of triethylamine (TEA) for esterification of the analytes at 60 °C led to a substantial conversion of citrate and *cis-*aconitate to itaconate (unpublished results). Since these metabolites occur in plasma in the micromolar range, this led to false positive detection of itaconate in the nanomolar range. Considering that separation of small organic acids by liquid chromatography would not require derivatization, we have thus chosen a sensitive high performance liquid chromatography—tandem mass spectrometry (HPLC-MS/MS) approach for the quantification of the three itaconate isomers. Over the course of the last few years several studies on itaconate and isomers reported targeted quantification methods using either hydrophilic interaction liquid chromatography (HILIC) [32,33] or reversed-phase ion-pairing chromatography [14,34,35,36,37,38]. Although HILIC seemed to be an obvious first choice to chromatographically separate small polar compounds, our preliminary studies revealed that it is less suited for organic acids in terms of peak shape, sensitivity, reproducibility after requilibration, and separation effect (unpublished results). This observation was recently confirmed by another study also proposing reversed-phase ion-pairing chromatography for itaconate and citraconate quantification [35]. Consequently, we chose to adapt a reversed-phase chromatography method that was previously established to separate citrate isomers and other organic acids of the TCA cycle [39,40]. This method accurately and reproducibly discriminates among the three itaconate isomers, shows no evidence of notable interisomeric conversion, and could be validated according to the US Food and Drug Administration (FDA) recommendations for bioanalytical method validation [41].

## 2. Results

### 2.1. Detection of Itaconate and Its Isomers Using HPLC-MS/MS

We intended to develop a sensitive quantification assay that included not only itaconate and its isomers but also its pharmacologically important derivatives 4-octyl itaconate and dimethyl itaconate as well as intermediate metabolites of the TCA cycle (Figure 1A). As mentioned above, in order to avoid interconversion among the target molecules we chose an HPLC-MS/MS method without derivatization, but featuring organic extraction with acetonitrile/methanol followed by thermally mild evaporation at 40 °C under nitrogen flow. Initially, we also included dimethyl itaconate among the analytes, but due to its comparatively low boiling point of 208 °C it evaporated during extraction of the samples and, therefore, could not be detected by this assay.

Since our assay also includes the quantification of various TCA metabolites (i.e., isocitrate, citrate, *cis-*aconitate, succinate, fumarate, malate, and lactate) which are quite abundant in human body fluids, such as plasma and whole blood, human plasma proved to be unsuitable as a matrix for the calibration curves. We therefore used pre-dialyzed human serum albumin (HSA, 50 g/L) in 1× phosphate buffered saline (PBS) as surrogate matrix for blood- and organ-based biosamples. Four stable isotope-labelled internal standards (ISTD) were added at the first step of organic sample extraction to correct for possible matrix effects and errors during sample preparation and measurements: ^13^C_3_-lactate, ^13^C_2_-citrate, ^13^C_6_-*cis-*aconitate, and ^13^C_5_-itaconate (in later preparations also ^13^C_4_-succinate, Figure 1C, analyte/ISTD pairs: Table S6).

Separation of analytes on a Shimadzu Nexera HPLC system was achieved on a Kinetex-C18 reversed phase column with water containing 0.2% (*v*/*v*) formic acid as mobile phase A and methanol containing 0.2% (*v*/*v*) formic acid as mobile phase B. While it may seem counterintuitive to further acidify the eluents, the addition of 0.2% formic acid actually improved ionization of the carboxylic acids. The total run time of the method was 11 min and the highly polar TCA metabolites eluted with the lowest retention times around 1.5–3 min, indicating very weak or no interaction with the stationary phase. The itaconate isomers eluted within 4.6–6.7 min in the order citraconate, mesaconate, itaconate and were baseline separated. The less polar 4-octyl itaconate eluted around 9 min and after applying the water-to-methanol gradient (Figure 1D, Table S5).

Limits of detection (LOD) and lower and upper limits of quantification (LLOQ, ULOQ) of the assay were determined with calibration curves prepared in surrogate matrix. According to FDA recommendations, LOD and LLOQ were defined as three times and five times, respectively, the response (peak area) of the matrix blank (Figure 1C, Tables S4 and S6). Individual calibrators were included for the final calibration curve fitting if the lowest calibrator exhibited <20% deviation and all other calibrators <15% of the expected concentration. Due to the presence of low residual concentrations of lactate, succinate, and citrate in the surrogate matrix even after repeated dialysis, LLOQ for these analytes were higher than for the other analytes. This resulted in at least five (lactate) and up to eleven (*cis-*aconitate, citraconate, itaconate, mesaconate) calibrators included in a quadratic regression with 1/x weighting. Values for LLOQ and ULOQ are listed in Table S6.

### 2.2. Validation of the Assay

The HPLC-MS/MS method was subsequently validated according to criteria of the FDA Guidelines for Bioanalytical Method Validation [41]. The results are summarized in Table 1 and Tables S6–S18. Quality control (QC) sets were prepared by spiking the analytes in defined concentrations into the surrogate matrix (Table 1). Unspiked surrogate matrix was used as matrix blank, and standard curves were prepared in surrogate matrix as described above. Intraday accuracy was determined by injecting one extracted QC set five times and calculating deviation from the expected concentration. The accuracy of all analytes at all QC levels was within 15% deviation of the expected concentration. To assess injection reproducibility of the measurements, intraday precision was determined in the same samples by computing the coefficient of variation (%CV); it varied by <5%CV for all analytes. Interday precision was determined using individually prepared QC sets of six measurement days (in total, *n* = 6 QC samples per level). All calculated %CV fulfilled the requirements, although %CV was >10 for 4-octyl-itaconate at QC-L and QC-M. Operator precision was examined by computing the %CV of 10 independently extracted samples of the same day with one injection per sample, which resulted in small deviations within the required range. The accuracy requirements for this validation step were missed by 4-octyl-itaconate at QC-L (119.3%) and also for TCA intermediates malate (QC-L: 116.6%) and succinate (QC-L: 78.5%; QC-M: 77.6%). Autosampler stability (overnight stability in the thermoelectrically cooled autosampler) was computed as the ratio of the mean concentration measured on day 1 with freshly prepared calibrators divided by the initial concentration measured on day 0; it ranged between 95–103% for all analytes at all three QC levels.

To assess the recovery of the analytes after sample extraction, QC sets and surrogate matrix blanks were extracted simultaneously. The extracted matrix blanks were then spiked with analyte concentrations at QCs levels, after which all samples were measured in batch. The response ratio pre/post- extraction was within the required range for all analytes except for 4-octyl-itaconate at QC-H (70.4% deviation). This high deviation pointed towards a systematic dilution error, since 4-octyl-itaconate was the only analyte pre-diluted in DMSO. Of note, there was no evidence of substantial interconversion of target molecules throughout sample extraction. Especially for itaconate isomers, the pre/post-extraction response percentage was nearly 100% at nanomolar and low micromolar concentrations (QC-L and QC-M).

Carryover of analytes was evaluated by measuring analyte response in double blanks (gradient-grade water) following QC-H samples and setting it in ratio with the analyte response at LLOQ. Only 4-octyl-itaconate (21.8%) exceeded the recommended maximal carryover of 20% LLOQ.

Since TCA intermediates and lactate are often highly concentrated in biosamples like plasma, the respective extracted samples need to be diluted to quantify the concentrations within the calibration range. To measure the impact of dilution on accuracy, QC-H samples were diluted 1:10 with gradient-grade water containing the internal standards at matching concentrations (Table S18). 

While the accuracy for itaconate isomers, 4-octyl-itaconate, and most TCA intermediates was within the recommended criteria, the measured concentrations for lactate, isocitrate and malate were generally too high (121–143%).

The above results showed that the assay could be fully validated according to FDA criteria for the quantification of itaconate, mesaconate, and citraconate, but only partially for 4-octyl-itaconate and TCA intermediates such as succinate. Malate and isocitrate eluted at the beginning of chromatography with all molecules and ions not interacting with the column and therefore were especially exposed to matrix effects such as ion suppression and, in case of dilution experiments, also to apparent ion enhancement that resulted from simultaneous dilution of the matrix.

### 2.3. Assessment of Preanalytic Biosample Parameters

The effects of repeated freezing-thawing cycles on measured concentrations and stability in extraction buffers, matrices, and biosamples are important preanalytic parameters for the evaluation and translational development of novel biomarkers.

#### 2.3.1. Freeze/Thaw Cycles

We subjected quality controls in surrogate matrix to up to four freezing-thawing cycles. After each cycle an aliquot was taken from each stock and extracted immediately. Concentrations of all analytes remained stable and within the recommended criteria, with the exception of the QC-L concentration of succinate, which was detected at up to 17.2% below input after the third freezing cycle (Table S11).

#### 2.3.2. Stability in Extraction Reagent and Stability of Dried Extracts

In most analytic approaches the prompt inactivation of biosamples to prevent later changes to the metabolic profile, e.g., by catalytic activities, is a major requirement. Besides snap freezing the samples in liquid nitrogen or on dry ice, direct organic extraction in acetonitrile/methanol/water 2:2:1 and subsequent storage at −20 °C seemed to be the most straightforward approach. Thus, the stability of all QC levels in organically extracted surrogate matrix was monitored for up to 35 days (Table S10) and the stability of dried extracts was assessed for three weeks (Table S11). The three itaconate isomers remained stable, although average accuracy of mesaconate at QC-M was marginally too high (117%) in dried extracts after three weeks. For the TCA intermediate succinate, the calculated concentrations in dried extracts were lower than acceptable (69–80%), while isocitrate, malate, and lactate concentrations were too high (for some QCs > 115%). This was also the case for fumarate in extracted samples stored at −20 °C.

#### 2.3.3. Stability in Human Plasma and Whole Blood

To assess the robustness of this assay to quantify itaconate, its isomers, and 4-octyl-itaconate in human blood and to test whether measurable catabolism occurs between collecting and freezing blood samples, we spiked these compounds into freshly prepared human plasma and whole blood from a healthy donor (donor C) and incubated plasma at room temperature (RT) or 4 °C for up to 24 h and whole blood at 37 °C for up to 8 h (Tables S14 and S15 for itaconate isomers and Tables S16 and S17). Baseline concentrations of all analytes were measured beforehand (Table S13). Of the itaconate isomers, only citraconate was quantifiable in plasma (0.39 µM) and whole blood (0.35 µM). Spiked-in itaconate remained stable at all QC concentrations, indicating that there was no significant catabolism or interconversion of itaconate by enzymes present in human plasma or whole blood. In contrast, mesaconate and citraconate could only be quantified reliably at nanomolar to low micromolar concentrations in plasma that was extracted directly after spike-in. At 40–80 µM spike-in concentrations, all itaconate isomers remained stable within the accuracy requirements for all time points. Unsurprisingly, recovery of most spiked-in TCA intermediates at comparably low QC-concentrations failed due to their high basal concentrations. Catalytic activities in the biosamples led to a dramatic shift in the metabolic profile over the course of incubation, leading e.g., to accumulation of lactate especially in whole blood (Table S17). These results underline the need for rapid sample processing in order to preserve the original metabolic profile of the TCA intermediates.

#### 2.3.4. Long Term Storage Stability of Unprocessed Samples

Biosamples such as frozen plasma and serum are stored in biobanks for months, years, and even decades. Thus, stability of analytes under these or similar conditions is of great interest. While samples in biobanks are usually stored at −80 °C or even colder, we subjected our QCs in surrogate matrix to −20 °C to allow for a first stability assessment of frozen samples after 6 months. Notably, all analytes remained stable for 6 months (Table S12) and although measured concentrations at QC-L were repeatedly too high for 4-octyl-itaconate (115.7–116.9% for 1 week–2 months) and in one case too low at QC-M (84.8% for 1 month), the measured concentrations in 6-month-old QCs were within the expected range for all analytes. This was most likely made possible by implementing ^13^C_4_-succinate as new internal standard for succinate and fumarate. For measurement of bench-top and 4 °C stability (Tables S7 and S8) we had observed that calculated succinate concentrations corrected by ^13^C_2_-citrate were constantly lower than expected, while concentrations of all other metabolites were well within the expected range or occasionally higher (isocitrate, lactate, malate). Since there was no apparent trend towards lower concentrations after longer incubation times, a systematic error was more likely. Therefore, we implemented ^13^C_4_-succinate as new internal standard for succinate and also for fumarate due to structural similarity and comparable retention time. Indeed, the accuracy of QC-H improved by 12.2% for succinate and by 7% for fumarate (Table S12).

### 2.4. Biological Proof-of-Concept Studies

#### 2.4.1. Detection in Human Whole Blood, Plasma, and Blood Leukocytes

We then applied the assay to assess the relative distribution of the isomers in different blood compartments, the effect of stimulation with LPS/IFNγ (mimicking a sepsis-like inflammation), and differences in concentration among various mouse organs. Since it has been reported that EDTA most likely interferes with LPS stimulation [42,43], while Li-heparin even enhances the binding of LPS to its receptor [44,45], we used Li-heparin blood for the initial experiment of whole blood stimulation with LPS/IFNγ.

**Figure 2:**
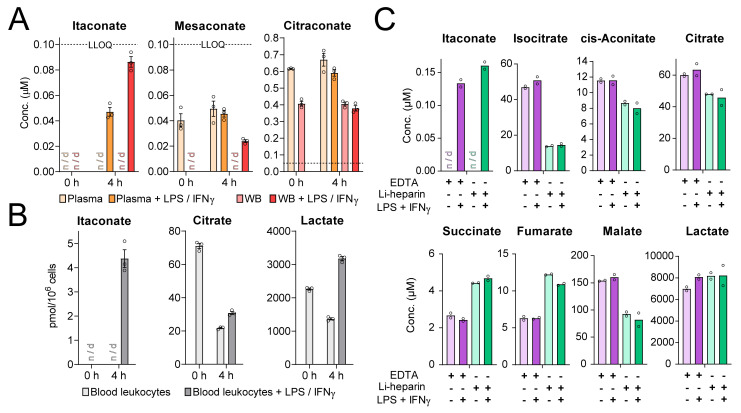
Itaconate levels in LPS/IFNγ−stimulated whole blood. (**A**)—Itaconate and mesaconate were detectable in whole blood (WB) and plasma after 4 h of stimulation, but could not be quantified reliably, since concentrations were <LLOQ. Citraconate levels were significantly higher, but not affected by LPS/IFNγ−stimulation. Plasma was obtained from Li-heparin anticoagulated blood collection tubes. Dashed line marks LLOQ. Samples from donor A; *n* = 3 aliquots from the same blood draw; mean ± SEM; n/d: not detected. Concentrations of TCA intermediates from this experiment are shown in Figure S1. (**B**)—Itaconate levels were quantifiable in blood leukocytes present in buffy coats from donor A upon LPS/IFNγ-stimulation; *n* = 3; mean ± SEM; n/d: not detected Citrate and lactate are shown to reveal the shift towards aerobic glycolysis. (**C**)—Comparison of TCA metabolite levels in EDTA and Li-heparin blood stimulated with LPS/IFNγ for 4 h. Itaconate was quantifiable in both preparations of stimulated blood, but mesaconate and citraconate levels were not detectable. TCA metabolite levels varied greatly when comparing EDTA and Li-heparin blood while citraconate and mesaconate levels were <LLOQ. Samples from donor B, *n* = 2 replicates from the same blood draw; mean; n/d: not detected.

In plasma and whole blood from donor A, only citraconate was detected above LLOQ and was apparently not affected by LPS/IFNγ stimulation (Figure 2A). Stimulation of whole blood for 4 h led to an apparent accumulation of itaconate, but the measured values were below LLOQ and did not allow assigning concentration values. Higher concentrations might have resulted if we had sampled later time points, since itaconate synthesis is initiated de novo by transcription of *ACOD1* mRNA during activation of monocytes and macrophages and likely had not reached maximum expression yet. We then tested whether the assay would detect a more pronounced induction of itaconate when we selectively analyzed only blood leukocytes present in buffy coats after erythrocyte lysis. Indeed, there was a strong itaconate peak 4 h after LPS/IFNγ stimulation, whereas mesaconate and citraconate concentrations were below LLOQ. This was accompanied by a drop in citrate and a rise in lactate (Figure 2B), which was consistent with a shift towards aerobic glycolysis under stimulation [46]. Finally, we assessed whether the type of anticoagulant in the blood collection tube (Li-heparin vs. EDTA) would affect measurements. In both cases, LPS/IFNγ stimulation led to a comparable induction of itaconate, but not mesaconate or citraconate. In contrast to the first experiment, citraconate was not detected in whole blood from donor B. Pronounced differences of EDTA and Li-heparin blood were detected in the measured concentrations of TCA intermediates in that at least isocitrate was more abundant in EDTA-blood and succinate and fumarate were more abundant in Li-heparin blood (Figure 2C).

#### 2.4.2. Detection in Mouse Organs

**Figure 3:**
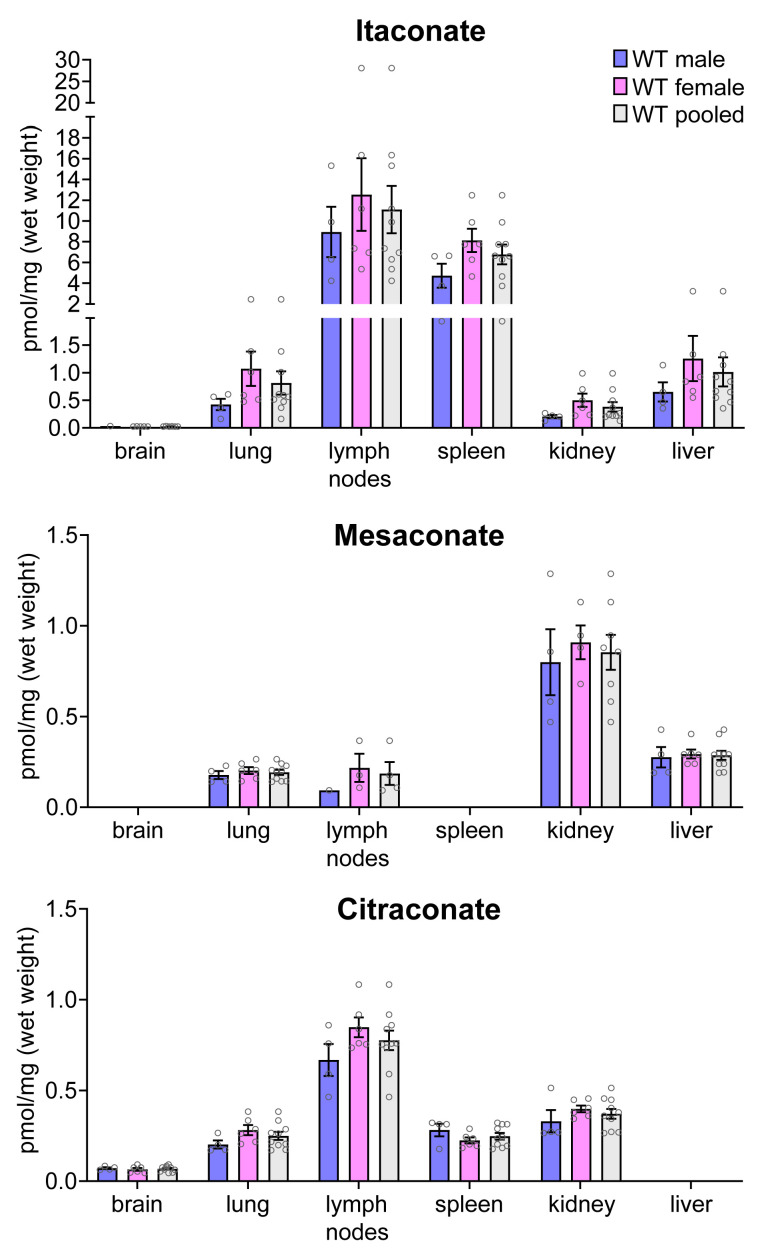
Itaconate, mesaconate, and citraconate concentrations in extracted mouse organs of 44 to 46 weeks old C57BL/6J wild-type (WT) mice. Itaconate was most abundant in organs related to the immune system while mesaconate was enriched in organs responsible for metabolism and secretion/excretion. Citraconate was also highest in lymph nodes and was the only isomer that could be quantified in brain; *n* = 4 male and *n* = 6 female mice; mean ± SEM; values < LLOQ were considered missing values.

A heterogeneous pattern emerged from the analysis of mouse organs prepared from aged (44–46 weeks old) C57BL*/6*J mice (Figure 3). As expected, itaconate concentrations were highest in immune organs (lymph nodes and spleen), whereas mesaconate was most abundant in kidney, and citraconate in lymph nodes. Only citraconate was detected in brain, albeit at very low concentrations. Overall, mesaconate and citraconate concentrations were roughly 10-fold lower than the highest itaconate concentrations, and citraconate levels in mouse organs were in the same range as the levels measured in human blood and plasma when normalized to tissue wet weight (mouse blood was not tested). There was no consistent correlation among concentrations of any two or all three isomers, and the differences in concentration between male versus female mice were not significant. Quantification of the TCA intermediates revealed a distinct profile for each organ (Figure S2).

## 3. Discussion

We have established and validated an HPLC-MS/MS assay for the detection of itaconate, mesaconate, and citraconate as well as lactate and selected TCA intermediates.

### 3.1. Potential Applications

This assay should find applications in the evaluation of the itaconate isomers as biomarkers for disease and treatment responses in humans and animals. Considering the ongoing efforts to develop itaconate and chemically modified variants thereof into anti-inflammatory medications, it should also prove useful to determine pharmacokinetics and -dynamics in preclinical studies. Remarkably, spike-in experiments revealed that itaconate remained stable even at low concentrations both in human plasma at RT and in whole blood at 37 °C. In contrast, concentrations of mesaconate, citraconate, and 4-octyl-itaconate changed over time when they were added in the nanomolar range. Most likely catabolism plays a role here, as the metabolic profile of the TCA intermediates also changed dramatically over time, especially in whole blood incubated at 37 °C.

Since mesaconate and 4-octyl-itaconate could not be quantified reliably in whole blood even after direct extraction when spiked-in at nanomolar and low micromolar concentration, an additional ion suppression effect by the complex matrix is likely. This observation will need to be taken into consideration when comparing administered doses against concentrations measured in blood, for instance in pharmacokinetic studies. Also, covalent binding to plasma proteins might affect mesaconate levels, as itaconate is known to undergo covalent binding to a variety of proteins containing free thiol groups [47] and the same may be true for mesaconate. It has been suggested that itaconate is catabolized inside of cells via itaconyl-CoA [48,49]. Our data suggest that this pathway is not active in plasma and that there also is no alternate catabolic mechanism in plasma that would reduce itaconate levels after phlebotomy.

The opportunity to quantify TCA intermediates at the same time is a particular strength of the assay, as increased succinate levels due to itaconate-mediated inhibition of SDH can be considered a measure of intracellular itaconate activity [10,25]. Although the assay could be validated for succinate concerning most of the basic criteria, subsequent stability tests revealed that correction by ^13^C_2_-citrate as ISTD might result in calculated concentrations being too low, an effect that became visible after recalibration of the mass spectrometer. This deviation could be corrected by introducing the internal standard ^13^C_4_-succinate, as demonstrated for the long-term stability test after 6 months. Due to similar structural properties and retention time, this internal standard should also be a better choice for fumarate.

Our results that itaconate could not be detected in normal plasma agree partially with those by Meiser et al., who showed (using a GC-MS assay) that itaconate could only be sporadically detected in plasma, serum, or urine from individuals with and without sepsis [17]. These authors argued that itaconate likely plays a major role inside the synthesizing cells but is not released into the extracellular environment. However, as shown in Table S1, there are three additional reports of detection of itaconate in human serum using GC-MS or HPLC-MS. One study reported an LLOQ of 0.5 µM for itaconate in serum, which is five times less sensitive than our method. The remaining two studies did not report absolute concentrations, and thus it is not possible to compare their sensitivities to our assay.

Recently, Tan and colleagues reported an alternative LC-MS/MS method for the quantification of itaconate and *cis-*aconitate [35]. While these authors showed that this assay separated itaconate and citraconate, they did not include mesaconate. Although the reported on-column LLOQ of 30 pg for itaconate was two-fold lower than ours (64 pg, Table S6), the essential per sample LLOQ was 20-fold higher and in the same range as the LLOQ of our assay. This was due to the focus on directly measuring methanol-extracted samples from 96-well plates. In addition, the reported on-column LLOQ for the second analyte, *cis-*aconitate, was 100 pg and therefore around 2.3-fold higher than the LLOQ reported in our study (43 pg). While the method by Tan and colleagues allows for rapid sample extraction, our method offers higher versatility in terms of sample types and sizes especially due to the evaporation step, which also enables concentration of samples.

### 3.2. Preanalytical Properties

Apart from the above-mentioned matrix effects on mesaconate and 4-octyl itaconate in plasma and whole blood, our study identified other important preanalytical properties of itaconate and its isomers. All three were stable in extraction buffer at −20 °C and as dried extracts for at least three weeks at RT. Generally speaking, the latter would reduce expenses and simplify logistics because sample extracts could be shipped at ambient temperature, at least when focusing on the quantification of the itaconate isomers.

We found that repeated freeze-thaw-cycles did not affect the measured concentrations of the included analytes, except succinate to a minor extent. However, the latter result was rather a consequence of the internal standard correction by ^13^C_2_-citrate than a stability issue and could most likely be corrected by ^13^C_4_-succinate. Thus, biobanked samples can be thawed and re-aliquoted or even refrozen for other studies unless other freeze-thaw-sensitive targets are to be measured in the same sample. 

The type of anti-coagulant (EDTA vs. Li-heparin), on the other hand, had strong effects on the measured concentrations of the TCA metabolites. This observation has been made before for profiling of amino acids and lipids [50] and it was suggested that ion suppression caused by EDTA is one of the reasons [51]. Thus, our results underscore existing knowledge that the same type of anti-coagulant needs to be used throughout one study and that anti-coagulant type needs to be documented for all biobanked samples.

### 3.3. Differences in Organ Distribution of Itaconate Isomers

Very little is known about distribution of itaconate and its isomers across human or animal organs. Citraconate and mesaconate have both been linked to inborn metabolic disorders that affect liver and/or kidney, and it is therefore no surprise that both were detected in kidney and mesaconate, in addition, in liver. Curiously, we could quantify citraconate in plasma (0.4–0.6 µM) and whole blood (0.35–0.4 µM) from two healthy donors (donors A and C), but did not detect it in whole blood from a third (donor B). Variable uptake from food or gut microbiota could explain such large differences, but the lack of detection of citraconate in mouse liver speaks against food as a major source. Clearly, further research is required to determine its internal and environmental sources and factors that govern its distribution in body fluids and organs.

The extensively documented functions of itaconate in the immune system are fully consistent with our observations that its highest concentrations were found in spleen and lymph nodes. Surprisingly, citraconate, too, was most abundant in lymph nodes, and a biologically relevant role in immunity would be intriguing and should be the subject of future studies. It should be kept in mind that the examined organs were obtained from healthy but relatively old (44–46 weeks) mice that were not kept under specific pathogen-free conditions. To our surprise, appreciable concentrations of itaconate were detected in lymph nodes and spleen even though there was no evidence of systemic inflammation. Further research is necessary to test whether increasing age and/or physiological low-grade immune reactions in mice can lead to concentrations in the range detected in our study. In any case, itaconate levels would be much higher in the context of systemic or severe local inflammation, which might also change the observed relative abundances in the examined organs.

In conclusion, we have developed and validated an HPLC-MS/MS assay which, according to regulatory requirements, enables the accurate quantification of itaconate and its isomers citraconate and mesaconate in human biosamples such as plasma. Concerning most FDA criteria, the method is also valid for the pharmacological derivative 4-octyl-itaconate and selected TCA intermediates. TCA intermediates with low retention times in this assay, especially malate and isocitrate, are prone to matrix effects and one should, therefore, be aware that heavy matrices might prohibit an accurate quantification of these analytes. Implementation of additional stable-isotope labelled internal standards for these TCA intermediates would likely improve accuracy in heavy matrices. An initial application of the assay revealed important preanalytic properties of the isomers and has begun to shed light on differences in relative distribution of itaconate isomers in human blood fractions and mouse organs. Thus, this study lays the foundation for further studies on itaconate and its isomers as potential biomarkers and pharmacological interventions.

## 4. Experimental Procedures

### 4.1. Reagents

Gradient-grade organic solvents and other chemicals included acetonitrile, methanol, and water (9017, 8402, 4218, J.T. Baker, Phillipsburg, NJ, USA), formic acid (56302, Honeywell/Fluka, Seelze, Germany) as well as DMSO (34869, Honeywell/Riedel de Haen, Seelze, Germany). Standard substances for method validation were obtained from Sigma-Aldrich, St. Louis, MO, USA (*cis-*aconitic acid: A3412, citric acid: 251275, citraconate: C82604, fumaric acid disodium salt: F1506, d-(+)-threo-isocitratic acid monopotassium salt: 58790, itaconate: I29204, l-lactic acid monolithium salt: L2250, l-(−)-malic acid: 112577, mesaconate: 131040, succinic acid disodium salt: W327700, ^13^C_2_-citric acid: 492078, ^13^C_4_-succinic acid: 491985), Toronto Research Chemicals, Toronto, Canada (^13^C_5_-itaconate: I931004, ^13^C_6_-*cis-*aconitic acid tripotassium salt: A189891, ^13^C_3_-lactic acid monosodium salt: L113507) and Cayman Chemical Company, Ann Arbor, MI, USA (4-octyl-itaconate: 25374). PBS (L-182-10, Biochrom AG, Berlin, Germany) and Plasbumin^®^ 20 (PZN: 05559812, Grifols, Barcelona, Spain) were used for surrogate matrix.

### 4.2. Preparation of Calibrations and QCs

In order to dilute residual TCA metabolites in Plasbumin^®^ 20 (HSA) to generate a suitable surrogate matrix, the solution was dialyzed against PBS made from gradient-grade water with a buffer-to-sample volume ratio of 1:100 and a 3500 Da molecular weight cutoff (Spectrum™ Spectra/Por™ 3 RC Dialysis Membrane Tubing, 132724, Fisher Scientific, Pittsburgh, PA, USA). After three buffer changes, the anticoagulant citrate was still detected in matrix blanks and additional dialysis did not lead to further improvement, which resulted in a higher LLOQ for this metabolite. This was also the case for lactate, whereas succinate seemed to be an impurity in the gradient-grade water, also resulting in a higher LLOQ. More recent experiments revealed that Milli-Q (Merck-Millipore, Burlington, MA, USA) purified water might not have this impurity.

All metabolites except 4-octyl-itaconate were dissolved in gradient-water to create concentrated stock solutions (250 mM for itaconate isomers and 750 mM for TCA intermediates). 4-octyl-itaconate was dissolved in DMSO (100 mM). These stock solutions were used to generate quality controls and calibrators in surrogate matrix at the given concentrations (Table 1, Table S6). Quality controls were aliquoted to 50 µL and were stored at −80 °C, −20 °C, 4 °C or room temperature for stability experiments or were directly extracted. Calibrator C11 (Table S6) was serially diluted 1:2 with surrogate matrix. The resulting calibrators C1–C11 and surrogate matrix blanks C0 were aliquoted to 50 µL and subsequently stored at −20 °C.

### 4.3. HPLC-MS/MS Assay

The assay was adapted from the previously published assay for analyzing TCA intermediates in cell extracts [39]. Samples and calibrators (50 µL) were extracted using methanol/acetonitrile/water (2/2/1, *v*/*v*, for standard extraction protocol: 1000 µL spiked with 0.1 µM ^13^C_2_-citrate and ^13^C_5_-itaconate, 0.2 µM ^13^C_6_-*cis-*aconitate and ^13^C_4_-succinate, as well as 1 µM ^13^C_3_-lactate as ISTD) in 2 mL safe-lock reaction tubes (0030120094, Eppendorf, Hamburg, Germany). Samples were vortexed for 30 s and frozen at −20 °C for at least 2 h to assure complete protein precipitation. After thawing, samples were centrifuged (10 min, 4 °C, 20.000× *g*) and supernatants were transferred to new 2 mL reaction tubes. Supernatants were evaporated under nitrogen flow while heating the samples to 40 °C in a metal block for 2 mL reaction tubes. The residuals were resuspended in 100 µL water, centrifuged (10 min, 4 °C, 20.000× *g*), and supernatants were transferred into MS vials with inserts (702284, 702732, Macherey-Nagel, Düren, Germany and 7615290, Labsolute, Höxter, Germany). If indicated, samples were diluted in gradient-grade water containing the internal standards at final sample concentration (usually 1 µM ^13^C_2_-citrate and ^13^C_5_-itaconate, 2 µM ^13^C_6_-*cis-*aconitate and ^13^C_4_-succinate and 10 µM ^13^C_3_-lactate).

Samples were then analyzed using a Nexera chromatography system consisting of a controller (CBM-20A), an autosampler (SIL-30AC), two pumps (LC-30AD), a degasser (DGU-20A5), and a column oven (CTO-20AC, Shimadzu, Japan), coupled to a QTRAP5500 triple quadrupole / linear ion trap mass spectrometer (Sciex, Framingham, MA, USA). Data acquisition and further quantification were performed using Analyst® Software 1.7 (Sciex, Framingham, MA, USA).

For chromatographic separation (injection volume: 10 µL), a Kinetex C18 column (dimensions: 100 Å, 100 × 3 mm, 2.6 µm, 00D-4462-Y0, Phenomenex, Torrance, CA, USA) was used which included a column saver (0.5 µm Filter, 55214-U, Supelco, Bellefonte, PA, USA) and C18 security guard (4 × 2.0 mm ID, AJO-4286, Phenomenex, Torrance, CA, USA). Analytes were eluted with 0.2% (*v*/*v*) formic acid in water (solvent A) and 0.2% (*v*/*v*) formic acid in methanol (solvent B), using the following gradient at a flow rate of 0.4 mL/min: 0–6.0 min: 1% B, 6.0–7.0 min: 1–90% B, 7.0–8.0 min: 90% B, 8.01–11.0 min 1% B. Autosampler and column oven temperatures were set to 4 °C and 30 °C, respectively. After chromatographic separation, analytes were ionized by electrospray ionization in negative ionization mode with the following instrument settings: curtain gas: 45, collision gas: medium, ion spray voltage: −4500 V, temperature: 400 °C, ion source gas 1/2: 60/75, dwell time: 30 ms per mass transition. Analyte-specific mass transitions and MS-parameters are shown in Table S5.

### 4.4. Quantification of Metabolites

The ratio of analyte peak area and ISTD peak area (also referred to as ISTD response ratio) was used for quantification. Values below limit of quantification (LLOQ) were considered missing values. For most analytes, the mass transition with the highest peak intensity was used for quantification, except for 4-octyl-itaconate, for which the peak with the second highest intensity was used for quantification, as the additional mass transitions ensured correct identification. Metabolite concentrations were calculated using a calibration curve, which was fitted with a quadratic regression weighted 1/x.

### 4.5. Human Blood Donors

Donors were recruited at Hannover Medical School and the TWINCORE Centre for Experimental and Clinical Infection Research (both Hannover, Germany). All participants provided informed written consent. Formal ethical review and approval were waived by the Ethics Committee of the State Board of Physicians of Lower Saxony due to low risk to participants.

### 4.6. Spike-In Experiments

Peripheral blood was drawn into K_3_EDTA coated S-Monovettes^®^ (7.5 mL: 01.1605.001, Sarstedt, Germany) from a single donor (donor C) and immediately pooled in 50 mL centrifuge tubes (62.547.254, Sarstedt, Germany). Whole blood samples were spiked with QC-L/M/H concentrations of all analytes using concentrated stock solution described above. Five 50 µl aliquots of each QC level and five 50 µL unspiked controls were extracted 15 min after spike-in and aliquotation and frozen at −20 °C as described above. For each QC level, the remaining spiked whole blood was carefully distributed over five wells of a 24-well plate and incubated at 37 °C/5% CO_2_. After 4 and 8 h, 50 µL aliquots from each well were extracted and frozen at −20 °C.

Plasma was prepared in 50 mL centrifuge tubes by centrifuging twice for 10 min at 2000× *g* and 4 °C. After each step, plasma was carefully transferred to a new centrifuge tube. Subsequently, plasma samples were spiked with QC-L/M/H concentrations of all analytes and aliquoted to 50 µL in 2 mL safe-lock reaction tubes. Five aliquots of each QC level and five 50 µL unspiked controls were immediately extracted and frozen at −20 °C. The remaining aliquots were incubated at room temperature and at 4 °C for up to 24 h and were subsequently extracted and frozen at −20 °C.

### 4.7. In Vitro Stimulation of Peripheral Whole Blood

For the initial experiment, peripheral blood was drawn in S-Monovettes^®^ containing Li-heparin coated beads (9 mL: 02.1065.001, Sarstedt, Germany) from a single donor (donor A). To compare anticoagulants in a second experiment, peripheral blood was collected in both S-Monovettes^®^ containing Li-heparin coated beads and S-Monovettes^®^ coated with K_3_EDTA (9 mL: 02.1066.001, Sarstedt, Germany), also from a single donor (donor B).

Whole blood was immediately pooled in 50 mL Falcon^®^ centrifuge tubes (352070, Corning, NY, USA) for each anticoagulant and further divided into untreated controls and samples co-stimulated with LPS (final concentration of 100 ng/mL, lipopolysaccharides from *Salmonella enterica* serotype typhimurium, L6511, Sigma-Aldrich, St. Louis, MO, USA) and recombinant human IFNγ (final concentration of 400 U/mL, 300-02, Peprotech, Rocky Hill, NJ, USA). Stimulated and unstimulated whole blood was incubated in cell culture flasks at 37 °C/5% CO_2_ for 4 h. Subsequently, 50 µL aliquots of whole blood were directly extracted as described above. For plasma preparation, 5 mL of each whole blood sample type were centrifuged twice in 15 mL Falcon^®^ centrifuge tubes (352095, Corning, NY, USA) at 2000× *g* and 4 °C for 10 min. Plasma was carefully removed, aliquoted to 50 µL, and extracted. The buffy coat layer was transferred to a new 15 mL centrifuge tube and incubated 10 min with red blood cell lysis buffer (11 814 389 001, Roche, Basel, Switzerland) according to the manufacturer’s protocol. The cell suspension was centrifuged at 500× *g* and 20 °C for 5 min, the supernatant was aspirated, the cells were carefully resuspended in 1x PBS, subsequently counted with a Scepter™ cell counter (Merck-Millipore, Burlington, MA, USA), aliquoted to 2–5 × 10^6^ cells and extracted.

### 4.8. Preparation and Extraction of Mouse Organs

All animal procedures were performed following guidelines from the Federation of European Laboratory Animal Science Associations. The study was approved by the regulatory authority of the German Federal State of Lower Saxony (Niedersächsisches Landesamt für Verbraucherschutz und Umwelt, LAVES, permit no. 33.4-42502-04-13/1281). 44 to 46 weeks old C57BL/6J mice were euthanized by CO_2_ asphyxiation, followed by immediate collection of brain, lung, spleen, lymph nodes, liver, and kidneys. The samples were immediately frozen on dry ice and subsequently stored at −80 °C until extraction.

Frozen organs were weighed in 2 mL FastPrep tubes filled with lysing matrix A (6910, MP Biomedicals, Santa Ana, CA, USA). Subsequently, sample weight was adjusted to 300 mg by addition of 1x PBS, and ice cold extraction reagent was then added (1.2 mL acetonitrile/methanol 1:1 and 0.1 mL acetonitril/methanol/water 2:2:1 containing internal standards). The organ samples were homogenized at 4 °C using a FastPrep^®^-24 Instrument (MP Biomedicals, Santa Ana, CA, USA) at a speed of 6.0 for 2 × 30 s. In between runs, the samples were cooled down again for 5 min. Subsequently, the samples were frozen at −20 °C for 24 h to enhance protein precipitation. After thawing, samples were centrifuged (10 min, 4 °C, 20.000× *g*) and 1.4 mL of the supernatants was carefully transferred to 2 mL reaction tubes without disturbing the pellet and aspirating the lipid layer. 50 µL calibrator aliquots in surrogate matrix were prepared in 2 mL reaction tubes by adding 250 µL 1x PBS and extraction reagent (1.2 mL acetonitrile/methanol 1:1 and 0.1 mL acetonitrile/methanol/water 2:2:1 containing internal standards) and transferring 1.4 mL of the supernatants after centrifugation. Sample extraction was conducted as described above.

## Figures and Tables

**Table 1:** Results of basic validation.

		Intraday			Interday			Operator			Autosampler Stability	Recovery	Carryover
	QC-L QC-M QC-H	5 Injections of One Sample Set			Independent Preparations on 6 Days			10 Preparations on One Day			24 h in Between Injections	Ratio Peak Area Pre-/Post Extraction (%)	Ratio Peak Area Blank/ LLOQ (%)
	Exp. Conc (µM)	Mean (µM)	Accuracy (%)	Precision (%CV)	Mean (µM)	Accuracy (%)	Precision (%CV)	Mean (µM)	Accuracy (%)	Precision (%CV)	Ratio Conc. d1/d0 (%)		
Itaconate	0.3	0.3	102.7	2.6	0.3	101.1	3.0	0.3	106.4	4.6	96.4	100.1	
	3	3.1	102.6	1.0	3.1	104.2	4.7	3.1	103.4	2.3	99.7	101.4	
	80	87.1	108.8	1.4	86.2	107.7	3.8	84.3	105.4	1.9	100.2	105.2	3.8 ± 5.1
Citraconate	0.15	0.2	100.7	4.0	0.2	100.7	6.7	0.2	114.5	4.6	90.5	103.8	
	3	3.1	102.9	2.4	3.1	103.7	5.2	3.0	101.0	2.8	102.3	101.0	
	40	44.0	110.0	1.2	43.4	108.4	3.8	42.9	107.2	2.1	98.2	106.3	5.1 ± 6.5
Mesaconate	0.3	0.3	107.7	1.8	0.3	104.1	4.9	0.3	105.4	7.0	95.2	99.2	
	3	3.1	104.9	2.6	3.2	105.4	4.8	3.1	102.9	1.5	97.9	101.7	
	80	86.9	108.7	1.4	86.1	107.6	3.4	85.2	106.5	2.1	99.0	106.1	4.5 ± 6.7
4-Octyl-Itaconate	0.03	0.03	110.9	2.2	0.03	107.9	12.2	0.04	119.3	6.8	96.6	88.8	
	0.3	0.3	90.5	2.3	0.3	95.4	10.6	0.3	99.0	2.3	96.1	114.9	
	2	2.3	112.7	1.9	2.3	113.8	5.2	2.1	107.1	3.0	97.3	170.4	21.8 ± 5.2
*cis-*Aconitate	0.15	0.2	100.5	1.3	0.1	99.9	4.8	0.1	98.1	4.1	102.6	99.3	
	3	3.1	102.5	0.9	3.1	102.8	4.0	3.0	101.5	2.0	98.8	101.3	
	40	43.7	109.2	1.6	43.5	108.8	2.9	42.5	106.3	1.4	99.3	109.2	3.3 ± 5
Citrate	1.2	1.3	105.3	0.8	1.3	105.4	1.1	1.3	104.8	3.8	101.3	94.9	
	6	6.4	106.2	1.3	6.4	106.0	2.0	6.2	103.5	1.0	101.0	97.2	
	20	22.0	109.9	2.1	21.8	108.9	3.8	20.9	104.7	1.4	99.8	104.4	0.2 ± 0.2
Fumarate	5	5.5	109.3	2.4	5.4	107.3	2.7	5.3	107.0	2.0	100.0	97.6	
	12	13.0	108.0	2.4	13.1	108.9	2.4	12.6	105.1	1.3	102.9	100.8	
	40	45.1	112.8	1.9	45.8	114.4	3.3	43.2	107.9	1.4	98.8	108.4	0.5 ± 1
Isocitrate	0.6	0.6	99.9	2.2	0.6	101.9	3.3	0.6	102.5	3.5	99.1	98.4	
	6	6.2	103.8	1.7	6.4	106.6	2.8	6.5	109.0	1.6	98.3	101.4	
	40	40.9	102.3	2.2	42.1	105.2	4.8	43.2	107.9	2.5	97.3	112.1	15.6 ± 11.3
Lactate	50	54.5	109.0	2.6	55.6	111.2	6.2	55.0	109.9	5.9	97.3	103.0	
	150	160.6	107.1	1.5	159.8	106.6	4.9	161.6	107.7	3.4	99.4	100.5	
	400	449.2	112.3	2.7	436.8	109.2	5.5	427.5	106.9	2.0	100.8	103.9	2.1 ± 1.3
Malate	0.6	0.7	109.8	3.8	0.7	110.4	4.1	0.7	116.6	3.9	97.9	99.8	
	6	6.4	107.5	2.1	6.6	109.3	3.2	6.7	111.5	2.0	98.3	102.5	
	40	41.4	103.4	2.0	42.0	104.9	4.9	43.0	107.5	2.3	99.7	110.7	18.8 ± 12.8
Succinate	5	4.6	92.5	1.4	4.4	87.6	7.0	3.9	78.5	2.7	98.6	99.1	
	12	11.3	94.2	2.0	10.8	90.0	6.1	9.3	77.6	1.7	102.2	101.5	
	40	41.2	103.0	1.7	39.7	99.2	6.6	35.3	88.3	1.9	97.8	106.5	13 ± 4.7

Deviations > 10% are underlined once; deviations > 15%, which do not meet FDA recommendations, are underlined twice.

## Data Availability

The data presented in this study are contained within the article.

## Supplementary Materials

The following are available online at https://www.mdpi.com/article/10.3390/metabo11050270/s1: Figure S1: TCA metabolite levels in LPS/IFNγ stimulated whole blood (WB) and plasma from the experiment shown in Figure 2A, Figure S2: TCA metabolite levels in mouse organs in extracted mouse organs of 44 to 46 weeks old C57BL/6J wild type (WT) mice from the experiment shown in Figure 3, Table S1: Summary of studies on itaconate quantification in diseases and disease models, Table S2: Summary of studies on mesaconate quantification in diseases and disease models, Table S3: Summary of studies on citraconate quantification in diseases and disease models, Table S4: Implementation of FDA recommendations for chromatographic assay validation, Table S5: Mass spectrometry parameters and HPLC retention times, Table S6: Limits of quantification for the HPLC-MS/MS assay, Table S7: Stability of quality controls in surrogate matrix at room temperature (bench-top), Table S8: Stability of quality controls in surrogate matrix at 4 °C (refrigerator), Table S9: Stability of quality controls in surrogate matrix processed with extraction solvent at −20 °C and −80 °C, Table S10: Stability of quality controls as dried extracts at RT, Table S11: Stability of quality controls in surrogate matrix after repeated freezing and thawing, Table S12: Long-term stability of quality controls in surrogate matrix at −20 °C, Table S13: Base level concentrations of itaconate isomers and TCA intermediates in plasma/whole blood from donor C, Table S14: Stability of spiked-in itaconate isomers and derivatives in plasma from donor C at RT and 4 °C, Table S15: Stability of spiked-in itaconate isomers and derivatives in whole blood from donor C at 37 °C, Table S16: Stability of spiked-in TCA metabolites in plasma from donor C at RT and 4 °C, Table S17: Stability of spiked-in TCA metabolites in whole blood from donor C at 37 °C, Table S18: Accuracy and precision of QC-H dilutions. References [52,53,54,55,56,57,58,59,60,61,62,63,64,65,66,67,68,69,70] are cited in the Supplementary Materials.

## Abbreviations

ACOD1: cis-aconitate decarboxylase 1; CAD, cis-aconitate decarboxylase; %CV, coefficient of variation; EDTA, ethylenediaminetetraacetic acid; FDA, US Food and Drug Administration; GC, gas chromatography; HILIC, hydrophilic interaction liquid chromatography; HPLC, high performance liquid chromatography; HSA, human serum albumin; IFNγ, interferon gamma; Irg1, immune responsive gene 1; ISTD, internal standard; LC, liquid chromatography; LPS, lipopolysaccharide; LLOQ, lower limit of quantification; LOD, limit of detection; MS, mass spectrometry; MS/MS, tandem mass spectrometry; NMR, nuclear magnetic resonance; PBS, phosphate buffered saline; QC-H, quality control high; QC-L, quality control low; QC-M, quality control medium; RT, room temperature; SDH, succinate dehydrogenase; TCA, tricarboxylic acid; ULOQ, upper limit of quantification; WB, whole blood; WT, wild type.
