# Supplementary material for: Establishment, Validation, and Initial Application of a Sensitive LC-MS/MS Assay for Quantification of the Naturally Occurring Isomers Itaconate, Mesaconate, and Citraconate
Source: Metabolites. 2021 Apr 26;11(5):270. doi: 10.3390/metabo11050270 (PMC8146994; doi:10.3390/metabo11050270)
Supplement: Supplementary file 1 [file metabolites-11-00270-s001.zip › metabolites-1153398-supplementary(1).pdf]

Supporting Information

Figures

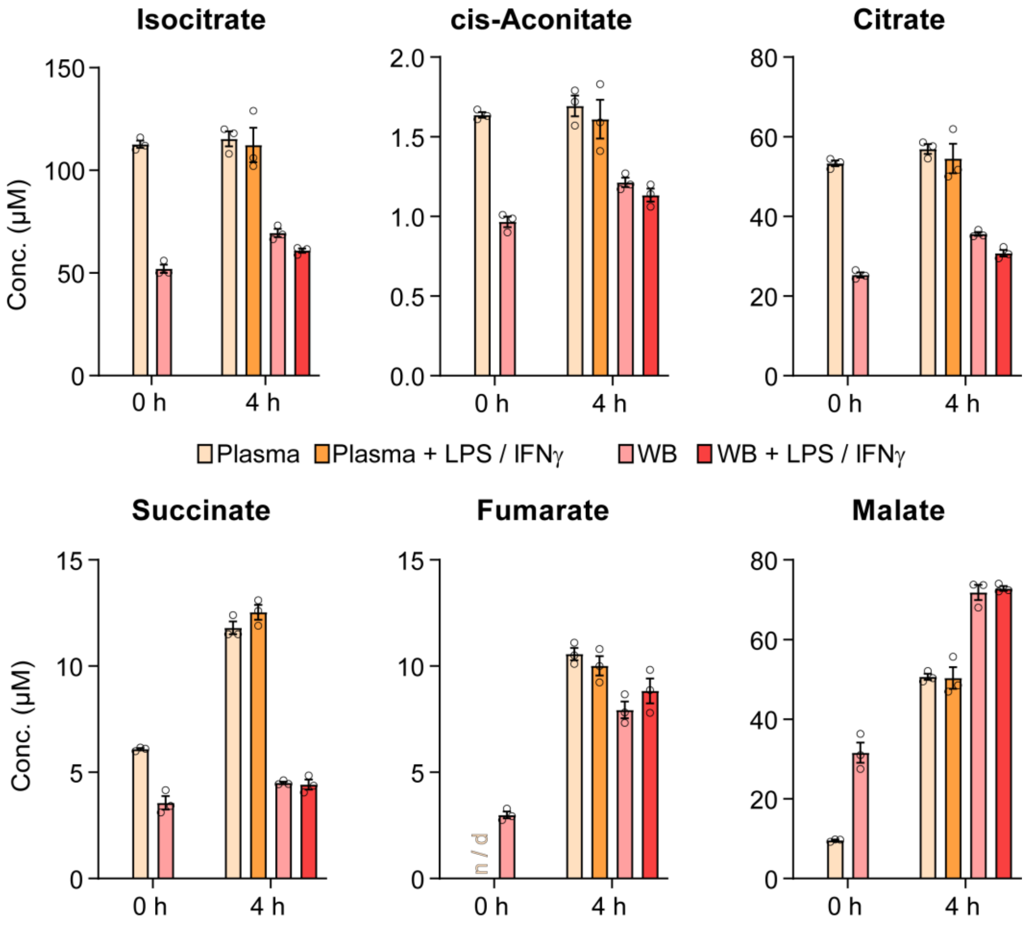

Figure S1. TCA metabolite levels in LPS/IFNγ stimulated whole blood (WB) and plasma from the experiment shown in Figure 2A**;** *n* =3; mean ± SEM; values < LLOQ were considered missing values. Lactate levels were > ULOQ. n/d = not detected.

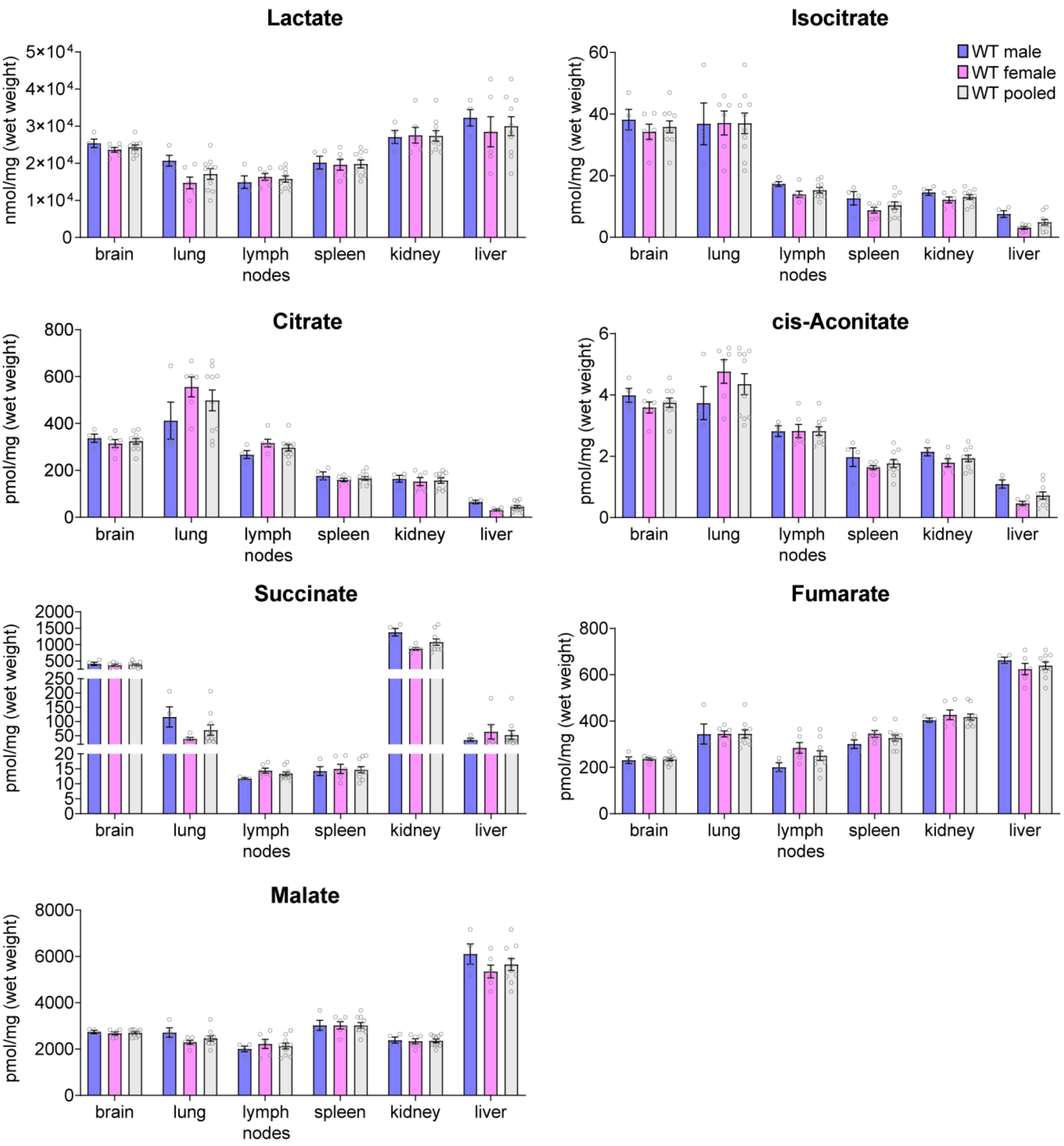

Figure S2. TCA metabolite levels in mouse organs in extracted mouse organs of 44 to 46 weeks old C57BL/6J mice from the experiment shown in Figure 3**;** *n* = 4 male and *n* = 6 female mice, mean ± SEM; values < LLOQ were considered missing values.

Tables

Table S1. Summary of studies on itaconate quantification in diseases and disease models.

Reference	Host	Disease/Model	Biosample	Method	Reported LLOQ
	**Human**				
[52]	Human	Sepsis	Serum	GC-MS	0.5 μM
[15]	Human	Systemic lupus erythematosus	Serum	HPLC-MS	–
[16]	Human<br>Aging female population	Overactive bladder syndrome	Urine	GC-MS	–
[17]	Human	Sepsis	Plasma, serum, broncho-alveolar lavage fluid, urine	GC-MS	1 μM (plasma)
[53]	Human	Gestational diabetes mellitus	Maternal serum at 20 weeks' gestation	GC-MS	–
	**Animal**				
[54]	Mouse	*P. aeruginosa* infection	Broncho-alveolar lavage	LC-MS	–
[13]	Chicken	*Eimeria acervulina* infection	Serum	UPLC-MS/MS	–
[55]	Mussel	*Vibrio* sp. infection	Tissue	GC-MS	6.25 μM
[12]	Mussel	*Vibrio* sp. infection	Tissue	GC-MS	–
[56]	Rat	Chronic fatigue syndrome (CFS)	Serum, urine	GC-MS	–
[57]	Mouse	Peritoneal tumors	Peritoneal macrophages	GC-TOF	–
[14]	Mouse	Rheumatoid arthritis	Hind limb extract, cell lysates (mouse synovial fibroblast culture),<br>K4IM cells (human synovial fibroblast cell line culture),<br>serum, urine	LC-MS/MS	–
[29]	Mouse	*Salmonella typhimurium* infection	Urine, serum, intestine, liver, spleen	NMR	–
[28]	Mouse	*Mycobacterium tuberculosis* infection	Lung	NMR	–

Table S2. Summary of studies on mesaconate quantification in diseases and disease models.

Reference	Host	Disease/Model	Biosample	Method	Reported LLOQ
	**Human**				
[58]	Human	Metabolomic changes following marathon (negative correlation with testosterone)	Serum	UHPLC-Q-TOF/MS and GC-TOF/MS	–
[23]	Human	Isovaleric acidemia	Urine	GC-MS	–
[24]	Human	Squalene synthase deficiency (defect in cholesterol biosynthesis; mesaconate is product of farnesol catabolism)	Urine	GC-MS	–
[59]	Human	IgA nephropathy	Urine	NMR	–
[60]	Human	Peritoneal dialysis patients with anemia/ intra-venous iron therapy	Serum	LC-MS	–
[61]	Human	Schizophrenia (mesaconate detected in healthy controls only)	Serum	NMR	–
[62]	Human	Treatment with sodium benzoate	Urine	GC	–
[63]	Human	Isovaleric academia, during a severe ketoacidotic attack	Urine	GC-MS	–
[64]	Human	Isovaleric acidemia	Urine	Headspace- GC	–
[65]	Human	Fasting and diabetic ketoacidosis (also citraconic acid)	Urine	GC	–
[66]	Human	Prenatal diagnosis of methylmalonic academia / isovaleric academia	Urine Serum	GC	–
	**Animal**				
[67]	Mice	Lung injury caused by paraquat	Lung tissues	LC-MS/MS	–
[68]	Rat	Feeding study methylsuccinate (rat)	Urine	GS-MS	–

Table S3. Summary of studies on citraconate quantification in diseases and disease models.

Reference	Host	Disease/Model	Biosample	Method	Reported LLOQ
	**Human**				
[69]	Human	*Porphyromonas gingivalis* infection in periodontal ligament cells (PDLSCs)	Periodontal ligament cells (PDLSCs)	GC-MS	–
[70]	Human<br>Mice	Extracellular magnesium deficiency	HepG2<br>RAW264.7<br>HUVECs	GC-MS	–
[20]	Human	Type 2 diabetes mellitus	Serum	GC/TOF-MS	–
[21]	Human	Polychlorinated biphenyls (PCB) exposure in maternal and cord serum	Serum (cord blood)	HILIC-MS/MS<br>GC-NICI-qMS	–
[22]	Human	Cirrhosis due to viral hepatitis vs. primary biliary cholangitis	Serum	NMR	–
[19]	Human	Colorectal cancer	Serum	LC-MS/MS	–
[18]	Human	Methylmalonyl-CoA mutase deficiency (deranged isoleucine metabolism / ketotic attacks / methylmalonic acidaemia)	Urine	GC-MS	–

Table S4. Implementation of FDA recommendations for chromatographic assay validation.

FDA Validation Recommendations for Chromatographic Assays (CCs)		Implementation for Assay Validation
**Parameter**	**Elements**	
Calibration Curve	- A blank (no analyte, no ISTD), a zero calibrator (blank plus ISTD), and at least six non-zero calibrator levels covering the quantitation range, including LLOQ in every run.<br>- All blanks and calibrators should be in the same matrix as the study samples.<br>- The concentration-response relationship should fit with the simplest regression model.	- 11 calibrator levels for Citraconate, Itaconate, Mesaconate & cis-Aconitate, 6-10 for the remaining analytes (**Table S6**).<br>- A surrogate matrix of PBS with 50 mg/ml HSA was used, due to the ubiquitous presence of TCA metabolites in most samples.<br>- the concentration-response relationship was fit with quadratic regression and 1/x weighting.
Quality Controls (QC)	- For A & P Runs: Four QCs, including LLOQ, low (L: defined as three times the LLOQ), mid M: defined as mid-range), and high (H: defined as high-range) from at least five replicates in at least three runs<br>- For other validation runs: L, M, andh QCs in duplicates	- Three QCs were included (L, M & H; **Table 1**) with at least five replicates for all validation runs.
Selectivity	- Analyze blank samples of the appropriate biological matrix from at least six individual sources.	- Base level concentrations of Itaconate isomers and TCA metabolites were analyzed in unspiked plasma and whole blood samples (**Table S13**).
Specificity	- The method specificity should be assessed for interference by cross-reacting molecules, concomitant medications, bio-transformed species, etc.	- All analytes were spiked in plasma and whole blood samples (base level concentrations in **Table S13**) at three QC concentrations (L, M & H; **Table S14/S15** for Itaconate isomers; **Table S16/S17** for TCA metabolites).
Carryover	- The impact of carryover on the accuracy of the study sample concentrations should be assessed.	- Carryover was monitored for each analyte in double blanks (HPLC grade water) following QC-H samples during HPLC-MS/MS sample analyses (measured in n=10 different batches; **Table 1**).
Sensitivity	- The lowest nonzero standard on the calibration curve defines the sensitivity (LLOQ).	- LLOQ (**Table S6**) was defined as five times the analyte response of the matrix blank according to recommendations.
Accuracy and Precision (A & P)	- A & P should be established with at least three independent A & P runs, four QC levels per run (LLOQ, L, M,h QC), and ≥ five replicates per QC level.	- A & P was established for intra-, interday and operator A & P runs (**Table 1**), three QC levels (L, M &H) per run and ≥5 (up to 10) replicates per QC level.
Recovery	- Extracted samples at L, M, andh QC concentrations versus extracts of blanks spiked with the analyte post extraction (at L, M, and H).	- Recovery experiment was performed with five replicates per QC level (L, M & H; **Table 1**).
Stability	- For auto-sampler, bench-top, extract, freeze-thaw, stock solution and long-term stability, perform at least three replicates at L andh QC concentrations.	- Each stability test was performed with five replicates, three QC levels (L, M &H) and up to four timepoints:<br>- Auto-sampler (**Table 1**),<br>- Bench-top (1-7 days at RT, **Table S7**)<br>- Fridge (1-21 days at 4 °C, **Table S8**)<br>- Extract (1-35 days in extraction solvent at -20 °C/-80 °C, **Table S9** & 1-21 days as dried extracts at RT, **Table S10**)<br>- Freeze/thaw (4 cycles, **Table S11**)<br>- Long-term stability (1-6 months at -20 °C, **Table S12**)
Dilution	- QCs for planned dilutions, five replicates per dilution factor.	- A & P of 1/10 diluted QC-H were analyzed for five replicates (**Table S18**).
Repeat Analysis	- No re-analysis of individual calibrators and QCs is permitted.	- Samples were only reanalyzed to assess autosampler stability (**Table 1**).

Table S5. Mass spectrometry parameters and HPLC retention times.

Analyte	Q1 Mass (Da)	Q3 Mass (Da)	Dwell Time (msec)	DP (volts)	CE (volts)	CXP (volts)	Retention Time (min)
**$^{13}C_5$-Itaconate_Q**	134.017	88.900	30	-25	-14	-5	6.75
**$^{13}C_5$-Itaconate_2**	134.017	44.100	30	-25	-22	-1	
**Itaconate/Citraconate_Q**	128.859	85.000	30	-30	-14	-7	Citra: 4.60
**Itaconate/Citraconate_2**	129.017	41.100	30	-25	-22	-1	Ita: 6.75
**Mesaconate_Q**	128.856	84.900	30	-30	-10	-9	5.85
**4-Octyl-itaconate-1**	240.949	111.000	30	-35	-18	-7	8.94
**4-Octyl-itaconate_Q**	240.949	66.900	30	-35	-30	-5	
**$^{13}C_6$-cis-Aconitate_Q**	178.870	134.000	30	-30	-12	-7	3.02
**$^{13}C_6$-cis-Aconitate_2**	178.870	115.900	30	-30	-12	-9	
**cis-Aconitate_Q**	172.938	85.051	30	-30	-16	-7	3.02
**cis-Aconitate_2**	172.938	110.878	30	-30	-12	-9	
**$^{13}C_2$-Citrate_Q**	192.909	112.951	30	-35	-18	-7	2.40
**$^{13}C_2$-Citrate_2**	192.909	87.958	30	-35	-24	-5	
**Citrate _Q**	190.886	110.984	30	-30	-18	-7	2.40
**Citrate_2**	190.886	86.968	30	-30	-22	-9	
**Fumarate_Q**	114.820	71.000	30	-45	-10	-7	2.78
**Isocitrate_Q**	190.909	110.832	30	-30	-20	-9	1.54
**Isocitrate_2**	190.909	73.019	30	-30	-30	-5	
**$^{13}C_3$-Lactate_Q**	91.829	45.000	30	-100	-16	-5	1.76
**$^{13}C_3$-Lactate_2**	91.829	43.000	30	-70	-34	-5	
**Lactate_Q**	88.890	43.096	30	-40	-18	-1	1.76
**Lactate_2**	88.890	45.018	30	-40	-16	-1	
**Malate_Q**	132.855	114.864	30	-30	-16	-7	1.49
**Malate_2**	132.855	70.931	30	-30	-22	-3	
**$^{13}C_4$-Succinate_Q**	121.000	76.000	30	-35	-16	-7	2.78
**$^{13}C_4$-Succinate_2**	121.000	103.000	30	-35	-14	-7	
**Succinate_Q**	116.847	73.000	30	-30	-16	-7	2.78
**Succinate_2**	116.847	99.000	30	-30	-14	-7	

DP, declustering potential; CE, collision energy; CXP, collision cell exit potential; Q, quantifier mass transition generally used for quantification.

Table S6. Limits of quantification for the HPLC-MS/MS assay.

Analyte	Internal Standard	Average LOD (µM)	Average LLOQ (Calibrator) (µM)	Average LLOQ per Sample (pmol)	Average LLOQ on Column (pmol)	Average LLOQ on Column (pg)	Average ULOQ (Calibrator) (µM)	Average R^2	Molar Mass (g/mol)
**Itaconate**	**$^{13}C_5$-Itaconate**	0.049	0.098 (C1)	4.90	0.490	64	100 (C11)	0.9999	130.099
**Citraconate**	**$^{13}C_5$-Itaconate**	0.025	0.049 (C1)	2.45	0.245	32	50 (C11)	0.9999	130.099
**Mesaconate**	**$^{13}C_5$-Itaconate**	0.049	0.098 (C1)	4.90	0.490	64	100 (C11)	0.9999	130.099
**4-Octyl-itaconate**	**$^{13}C_5$-Itaconate**	0.003	0.020 (C3)	1.00	0.100	24	5 (C11)	0.9999	242.312
**cis-Aconitate**	**$^{13}C_6$-cis-Aconitate**	0.025	0.049 (C1)	2.45	0.245	43	50 (C11)	1.0000	174.109
**Citrate**	**$^{13}C_2$-Citrate**	(0.195)*	0.391 (C5)	19.55	1.955	376	25 (C11)	0.9998	192.124
**Fumarate**	**($^{13}C_2$-Citrate) $^{13}C_4$-Succinate**	0.780	1.560 (C6)	78.00	7.800	905	50 (C11)	1.0000	116.072
**Isocitrate**	**$^{13}C_3$-Lactate**	0.012	0.098 (C2)	4.90	0.490	94	50 (C11)	0.9999	192.124
**Lactate**	**$^{13}C_3$-Lactate**	(7.8-15.6)*	31.300 (C7)	1565.00	156.500	14097	500 (C11)	0.9998	90.078
**Malate**	**$^{13}C_3$-Lactate**	0.049	0.196 (C3)	9.80	0.980	131	50 (C11)	0.9999	134.088
**Succinate**	**($^{13}C_2$-Citrate) $^{13}C_4$-Succinate**	(0.391)*	0.781 (C5)	39.05	3.905	461	50 (C11)	0.9998	118.088

Calibrators range from C0 (matrix blank) to C11; LOD: limit of detection; LLOQ and ULOQ: lower and upper limit of quantification; R^2 of quadratic regression with 1/x weighting. *Due to residual impurities in the surrogate matrix, the LOD for these analytes is specified as > 3× the signal (peak area) of the matrix blank.

Table S7. Stability of quality controls in surrogate matrix at room temperature (bench-top).

QCs in Surrogate Matrix at RT for	Exp. Conc. (µM)	Mean (µM)	Accuracy (%)	Precision (%CV)	Mean (µM)	Accuracy (%)	Precision (%CV)	Mean (µM)	Accuracy (%)	Precision (%CV)
		1 day			3 days			1 week		
Itaconate	0.3	0.3	104.1	1.9	0.3	102.7	1.3	0.3	103.0	2.9
	3	3.2	105.8	1.4	3.2	106.1	0.7	3.2	106.8	1.8
	80	73.3	91.6	1.6	74.7	93.3	2.0	80.9	101.1	3.5
Citraconate	0.15	0.2	109.5	3.0	0.2	109.7	2.2	0.2	111.2	2.6
	3	3.2	105.7	1.8	3.2	105.4	1.2	3.2	106.9	1.5
	40	39.3	98.2	1.5	39.5	98.8	1.6	41.6	104.1	2.4
Mesaconate	0.3	0.3	101.5	2.7	0.3	101.5	2.1	0.3	100.8	4.2
	3	3.3	109.3	1.0	3.3	109.4	0.5	3.3	110.7	3.0
	80	78.8	98.5	1.4	79.3	99.2	1.7	82.5	103.2	2.3
4-Octyl-itaconate	0.03	0.03	86.1	7.3	0.03	90.9	10.2	0.03	95.5	8.3
	0.3	0.3	101.3	5.0	0.3	101.1	2.4	0.3	106.5	4.1
	2	1.8	91.4	1.5	1.8	91.0	1.0	1.9	94.6	2.3
cis-Aconitate	0.15	0.1	91.5	4.7	0.1	93.9	7.0	0.1	92.9	4.1
	3	2.9	96.7	1.7	2.9	96.9	0.9	2.9	96.7	2.0
	40	37.7	94.3	1.5	37.9	94.8	1.4	39.9	99.8	1.3
Citrate	1.2	1.3	107.2	1.7	1.3	105.3	1.2	1.3	104.3	1.9
	6	6.4	105.9	1.2	6.4	106.5	5.0	6.3	104.6	1.0
	20	19.8	99.0	2.2	19.6	98.0	1.7	20.1	100.7	1.2
Fumarate	5	5.1	102.6	1.2	5.2	103.0	1.3	5.3	106.1	1.4
	12	12.6	104.7	4.2	12.8	106.5	2.8	13.3	111.2	2.0
	40	35.6	89.1	2.6	37.0	92.5	2.2	41.9	104.7	5.7
Isocitrate	0.6	0.7	115.2	3.2	0.7	109.4	1.3	0.6	101.0	2.0
	6	7.3	122.4	2.9	7.1	117.6	2.8	6.6	110.4	1.7
	40	43.3	108.3	1.4	42.9	107.2	2.1	40.9	102.3	3.8
Lactate	50	48.7	97.4	8.3	47.6	95.1	2.3	50.1	100.2	4.0
	150	159.2	106.1	2.1	160.0	106.7	1.7	167.8	111.9	3.2
	400	409.0	102.3	0.9	413.0	103.3	1.6	423.2	105.8	1.8
Malate	0.6	0.7	117.6	3.8	0.7	114.2	3.0	0.7	108.6	2.1
	6	7.2	119.2	3.2	7.1	117.6	3.0	6.9	115.0	5.7
	40	43.4	108.5	1.1	43.1	107.7	1.8	41.9	104.7	2.8
Succinate	5	3.4	68.1	0.7	3.5	70.0	2.8	3.6	72.1	3.2
	12	8.9	73.9	4.3	9.0	75.3	1.9	9.5	79.3	1.1
	40	27.0	67.4	2.5	27.7	69.4	1.7	30.8	77.0	4.4

Deviations >10% are underlined once; deviations >15%, which do not meet FDA recommendations, are underlined twice; *n* = 5 for each QC and time point.

Table S8. Stability of quality controls in surrogate matrix at 4 °C (refrigerator).

QCs in Surrogate Matrix at 4 °C for	Exp. Conc. (μM)	Mean (μM)	Accuracy (%)	Precision (%CV)	Mean (μM)	Accuracy (%)	Precision (%CV)	Mean (μM)	Accuracy (%)	Precision (%CV)	Mean (μM)	Accuracy (%)	Precision (%CV)
		1 day			1 week			2 weeks			3 weeks		
Itaconate	0.3	0.3	103.1	2.0	0.3	101.2	1.8	0.3	99.6	2.9	0.3	102.7	5.2
	3	3.2	105.7	2.8	3.2	105.2	0.8	3.2	105.1	2.0	3.2	105.8	1.5
	80	77.0	96.3	1.7	80.8	101.0	1.8	79.9	99.9	1.3	79.2	99.0	4.4
Citraconate	0.15	0.2	109.2	4.3	0.2	107.1	3.9	0.2	106.3	3.1	0.2	108.7	4.5
	3	3.1	104.7	2.7	3.2	105.1	2.3	3.2	105.7	2.2	3.2	105.7	0.7
	40	40.1	100.3	1.0	41.0	102.6	1.1	41.0	102.5	0.9	40.7	101.9	2.2
Mesaconate	0.3	0.3	102.2	3.0	0.3	104.5	2.5	0.3	104.3	3.7	0.3	104.1	4.1
	3	3.3	109.1	2.5	3.3	109.5	1.8	3.3	110.1	1.8	3.3	109.9	2.1
	80	79.9	99.9	1.7	80.8	101.0	1.0	80.6	100.8	1.5	80.9	101.1	1.7
4-Octyl-itaconate	0.03	0.03	100.5	12.2	0.03	108.3	5.6	0.03	110.3	2.5	0.03	113.1	3.0
	0.3	0.3	102.3	4.7	0.3	102.5	4.1	0.3	105.2	2.9	0.3	102.7	3.0
	2	1.9	93.5	1.9	1.8	89.1	7.7	1.9	96.7	2.2	1.9	95.0	4.3
cis-Aconitate	0.15	0.1	95.3	6.8	0.2	109.6	2.3	0.2	105.7	1.1	0.2	107.7	2.6
	3	2.9	97.0	1.9	3.0	98.5	2.9	2.9	97.1	1.0	2.9	97.2	1.4
	40	38.4	96.1	1.3	39.9	99.7	1.1	39.3	98.2	1.5	39.6	98.9	3.4
Citrate	1.2	1.3	105.7	0.9	1.3	107.2	2.0	1.3	108.2	4.0	1.4	113.3	8.9
	6	6.4	106.0	1.6	6.3	104.9	1.7	6.4	106.9	0.9	6.4	106.9	0.6
	20	20.5	102.5	1.5	20.4	102.1	1.1	20.0	99.9	2.1	20.3	101.6	3.0
Fumarate	5	5.1	102.4	1.6	5.4	107.5	1.9	5.3	106.5	3.1	5.4	107.4	3.2
	12	12.4	103.3	2.4	13.2	109.7	1.7	13.2	110.2	1.1	13.1	109.2	3.3
	40	38.4	96.1	2.1	41.4	103.6	3.8	40.1	100.4	5.8	40.4	101.1	8.9
Isocitrate	0.6	0.7	113.8	2.6	0.6	104.9	1.8	0.6	103.5	3.5	0.6	106.2	5.6
	6	7.4	123.9	2.4	6.7	111.4	1.0	6.7	111.9	1.9	6.8	112.8	5.2
	40	42.8	106.9	0.6	41.5	103.8	2.1	41.3	103.2	4.4	41.0	102.5	2.6
Lactate	50	49.3	98.5	3.7	48.7	97.4	3.4	54.9	109.8	6.6	58.3	116.6	9.4
	150	159.0	106.0	1.4	162.4	108.3	1.9	167.8	111.9	1.7	167.0	111.3	1.6
	400	409.4	102.4	1.6	427.4	106.9	1.3	426.8	106.7	1.3	423.6	105.9	2.0
Malate	0.6	0.7	115.1	2.5	0.7	111.5	5.2	0.7	111.8	6.2	0.7	115.0	8.1
	6	7.2	119.9	2.7	6.7	112.2	1.4	6.8	112.8	1.4	6.8	113.3	5.3
	40	42.5	106.4	0.8	42.0	104.9	2.1	41.7	104.3	3.4	41.6	104.0	2.4
Succinate	5	3.5	69.2	2.1	3.6	72.8	1.8	3.8	76.8	1.6	3.9	77.2	2.0
	12	8.8	73.2	1.9	9.4	78.0	2.3	9.7	80.5	0.6	9.4	78.6	4.0
	40	28.7	71.8	2.7	30.4	76.0	3.5	30.4	76.0	5.5	29.9	74.7	7.8

Deviations >10% are underlined once; deviations >15%, which do not meet FDA recommendations, are underlined twice; *n* = 5 for each QC and time point.

Table S9. Stability of quality controls in surrogate matrix processed with extraction solvent at −20 °C and −80 °C.

QCs in Extraction Solvent at −20 °C for	Exp. Conc. (µM)	Mean (µM)	Accuracy (%)	Precision (%CV)	Mean (µM)	Accuracy (%)	Precision (%CV)	Mean (µM)	Accuracy (%)	Precision (%CV)	Mean (µM)	Accuracy (%)	Precision (%CV)	Mean (µM)	Accuracy (%)	Precision (%CV)
		1 day			2 days			7 days			35 days			21 days at −80 °C		
Itaconate	0.3	0.3	103.9	2.4	0.3	97.6	4.9	0.3	102.7	2.5	0.3	107.3	2.3	0.3	109.8	1.6
	3	3.1	102.1	1.5	3.0	101.4	2.1	3.2	105.2	1.7	3.2	105.1	0.5	3.3	108.9	1.8
	80	87.7	109.6	3.5	84.2	105.3	2.6	84.6	105.7	2.1	85.3	106.7	1.4	87.9	109.9	2.3
Citraconate	0.15	0.2	103.1	2.3	0.1	96.1	5.6	0.2	102.9	5.1	0.2	101.2	5.8	0.1	96.1	5.5
	3	3.0	100.6	3.2	3.1	101.7	2.9	3.1	103.6	1.3	3.0	101.5	2.3	3.2	106.5	1.5
	40	43.5	108.8	3.8	42.2	105.5	1.8	42.3	105.8	1.7	43.1	107.8	1.6	43.1	107.8	3.0
Mesaconate	0.3	0.3	97.7	7.8	0.3	106.1	4.3	0.3	105.7	2.8	0.3	105.8	4.9	0.3	110.5	4.5
	3	3.1	102.7	2.2	3.1	104.5	2.2	3.2	106.3	0.6	3.1	103.7	2.3	3.2	105.8	0.9
	80	87.3	109.2	3.9	85.2	106.5	1.8	84.3	105.4	2.1	86.0	107.5	1.3	86.7	108.4	2.0
4-Octyl-itaconate	0.03	0.03	106.2	5.6	0.03	88.9	5.8	0.03	114.9	1.8	0.03	97.0	8.8	0.03	103.8	10.9
	0.3	0.3	88.0	2.9	0.3	85.3	4.1	0.3	103.4	1.3	0.2	77.0	0.8	0.2	82.8	9.5
	2	2.0	99.3	3.6	1.8	92.0	2.5	2.2	111.8	1.6	1.7	85.0	0.4	2.0	101.5	9.8
cis-Aconitate	0.15	0.2	102.5	3.8	0.2	101.9	4.0	0.1	98.3	6.0	0.2	100.1	2.7	0.1	98.4	3.6
	3	3.0	100.4	2.1	3.1	102.2	1.4	3.1	104.3	1.4	3.1	103.2	2.5	3.2	105.3	2.2
	40	43.3	108.4	1.4	43.1	107.8	2.0	44.1	110.2	1.7	43.9	109.8	2.0	44.3	110.8	2.2
Citrate	1.2	1.3	105.7	1.8	1.3	104.7	1.8	1.3	112.0	2.5	1.4	113.7	1.7	1.3	107.7	2.1
	6	6.3	105.7	2.4	6.6	109.6	1.5	6.6	110.2	2.4	6.9	114.7	1.1	6.6	110.1	1.1
	20	21.5	107.3	1.7	22.0	110.2	2.0	21.9	109.4	1.1	23.5	117.5	2.4	22.0	110.2	2.1
Fumarate	5	5.4	108.8	1.9	5.4	108.8	2.9	5.7	114.6	1.9	5.9	117.2	1.0	5.6	112.3	4.3
	12	12.7	106.2	1.8	13.0	108.3	2.9	13.7	113.8	2.3	14.1	117.2	3.2	14.0	116.5	2.5
	40	44.0	110.0	2.7	44.8	112.0	3.0	46.5	116.2	2.6	47.7	119.4	3.3	45.3	113.3	2.0
Isocitrate	0.6	0.6	98.3	5.0	0.6	101.1	3.2	0.6	95.9	2.7	0.6	106.6	4.7	0.7	108.6	5.4
	6	6.3	104.2	2.7	6.5	108.4	3.2	6.2	104.0	2.2	6.4	107.4	2.4	6.9	115.3	3.9
	40	41.4	103.5	3.2	40.1	100.3	2.0	40.8	102.0	4.4	43.3	108.3	2.8	43.8	109.4	0.9
Lactate	50	52.2	104.3	2.1	56.4	112.8	11.0	58.3	116.6	11.4	52.1	104.3	6.6	54.2	108.4	6.2
	150	158.0	105.3	4.0	161.8	107.9	2.7	165.8	110.5	1.3	163.0	108.7	4.1	171.6	114.4	5.1
	400	432.8	108.2	2.9	427.2	106.8	2.2	445.4	111.4	1.5	442.4	110.6	3.9	441.4	110.4	1.5
Malate	0.6	0.6	107.1	3.9	0.7	111.9	3.1	0.7	110.7	4.5	0.7	108.4	3.1	0.7	118.5	6.0
	6	6.4	107.2	2.9	6.7	111.5	1.6	6.5	107.5	2.5	6.6	110.1	2.0	7.2	120.1	3.4
	40	42.0	105.1	2.9	40.5	101.2	1.9	41.0	102.4	3.6	44.3	110.7	2.8	45.2	113.0	1.4
Succinate	5	4.4	88.0	2.2	4.5	89.2	3.3	4.6	92.3	2.7	4.6	91.4	3.4	4.5	89.8	5.9
	12	10.6	88.3	2.4	11.1	92.5	0.9	11.1	92.8	2.9	11.1	92.7	2.7	11.2	93.3	3.0
	40	38.3	95.9	3.2	39.9	99.7	3.0	40.3	100.9	2.5	42.4	106.1	2.5	40.0	100.0	2.1

Deviations >10% are underlined once; deviations >15%, which do not meet FDA recommendations, are underlined twice; n = 5 for each QC and time point.

Table S10. Stability of quality controls as dried extracts at RT.

Dried Extracts Stability at RT for	Exp. Conc. (µM)	Mean (µM)	Accuracy (%)	Precision (%CV)	Mean (µM)	Accuracy (%)	Precision (%CV)	Mean (µM)	Accuracy (%)	Precision (%CV)	Mean (µM)	Accuracy (%)	Precision (%CV)
		1 day			1 week			2 weeks			3 weeks		
Itaconate	0.3	0.3	101.8	2.6	0.3	101.7	1.8	0.3	102.2	6.3	0.3	102.2	2.7
	3	3.2	105.3	1.5	3.1	103.9	1.8	3.2	105.3	1.3	3.2	107.4	1.2
	80	78.6	98.2	1.3	73.4	91.8	3.6	77.4	96.7	1.4	83.3	104.1	3.1
Citraconate	0.15	0.2	113.7	4.9	0.2	105.2	4.4	0.2	109.5	1.0	0.2	111.3	4.3
	3	3.1	104.8	1.6	3.1	103.7	1.4	3.2	105.7	1.4	3.3	109.3	2.8
	40	40.7	101.9	1.3	38.8	97.0	3.2	40.6	101.6	1.1	42.5	106.4	1.8
Mesaconate	0.3	0.3	100.3	5.9	0.3	107.8	2.6	0.3	108.1	7.4	0.3	105.9	7.1
	3	3.3	109.0	1.6	3.2	107.5	1.3	3.3	109.0	1.6	3.5	117.1	2.9
	80	81.2	101.5	1.6	77.9	97.3	2.7	80.3	100.4	1.0	83.1	103.8	2.0
4-Octyl-itaconate	0.03	0.03	98.7	11.8	0.03	93.3	15.3	0.03	93.9	11.2	0.03	95.7	4.5
	0.3	0.3	103.5	2.2	0.3	91.7	7.5	0.3	93.3	3.2	0.3	96.6	3.5
	2	1.9	95.7	2.7	1.7	82.8	6.2	1.8	88.0	1.6	1.8	89.0	3.4
cis-Aconitate	0.15	0.1	94.1	7.1	0.2	107.6	1.8	0.2	108.7	4.1	0.2	106.4	3.7
	3	2.9	97.1	1.6	2.9	96.3	0.5	2.9	97.7	1.6	3.0	100.5	1.2
	40	39.6	99.0	0.8	40.1	100.2	4.9	39.0	97.6	1.8	41.0	102.6	2.2
Citrate	1.2	1.3	105.2	4.5	1.3	107.8	2.3	1.3	110.5	6.7	1.4	118.2	2.5
	6	6.3	104.8	1.7	6.5	107.8	0.8	6.4	106.6	1.4	6.8	113.5	7.2
	20	20.4	101.8	1.8	20.0	100.0	1.5	20.3	101.7	1.8	22.1	110.3	7.8
Fumarate	5	5.1	101.0	3.0	5.0	100.2	2.7	5.2	103.4	3.2	5.3	105.8	1.9
	12	12.3	102.7	1.9	12.3	102.7	2.5	12.5	103.8	3.3	13.1	108.8	1.3
	40	40.7	101.7	3.7	36.9	92.2	4.4	37.0	92.5	4.1	41.8	104.4	7.8
Isocitrate	0.6	0.7	112.6	3.0	0.7	115.7	3.6	0.7	114.3	4.1	0.6	106.9	2.1
	6	7.2	120.7	2.4	7.3	121.9	4.2	7.4	123.3	2.6	6.8	114.1	2.1
	40	39.8	99.6	3.4	41.1	102.7	5.3	42.9	107.3	1.4	42.4	106.1	3.9
Lactate	50	55.3	110.5	3.6	52.8	105.6	4.3	56.0	112.0	7.7	67.1	134.2	20.4
	150	162.4	108.3	2.8	165.8	110.5	2.5	168.2	112.1	4.0	186.6	124.4	11.8
	400	403.6	100.9	2.0	411.0	102.8	1.9	425.2	106.3	1.2	467.4	116.9	10.6
Malate	0.6	0.7	119.6	10.5	0.8	133.4	6.8	0.7	122.8	4.9	0.7	114.7	3.0
	6	7.1	117.5	2.4	7.4	123.9	6.7	7.5	125.2	4.1	7.0	116.9	2.8
	40	40.1	100.2	3.3	41.9	104.8	4.3	43.8	109.5	1.2	42.3	105.7	5.4
Succinate	5	3.5	69.6	3.4	3.7	73.5	1.7	3.8	75.3	4.0	3.8	76.9	2.6
	12	8.8	73.5	2.5	9.1	76.2	2.4	9.2	76.8	1.3	9.7	80.8	1.7
	40	30.3	75.7	4.3	28.2	70.4	2.1	27.6	69.0	3.4	30.6	76.5	6.4

Deviations >10% are underlined once; deviations >15%, which do not meet FDA recommendations, are underlined twice; $n = 5$ for each QC and time point.

Table S11. Stability of quality controls in surrogate matrix after repeated freezing and thawing.

Freeze/Thaw Cycles	Exp. Conc. (µM)	Mean (µM)	Accuracy (%)	Precision (%CV)	Mean (µM)	Accuracy (%)	Precision (%CV)	Mean (µM)	Accuracy (%)	Precision (%CV)	Mean (µM)	Accuracy (%)	Precision (%CV)
		1 cycle			2 cycles			3 cycles			4 cycles		
Itaconate	0.3	0.3	106.8	2.4	0.3	101.4	4.2	0.3	100.7	2.0	0.3	98.6	4.4
	3	3.1	101.7	0.9	3.0	100.6	1.2	3.0	98.4	0.9	3.1	103.2	1.4
	80	85.2	106.5	0.7	83.0	103.7	1.3	83.0	103.8	1.6	82.4	103.0	1.1
Citraconate	0.15	0.1	99.2	1.9	0.2	100.9	1.9	0.2	102.8	5.3	0.1	96.8	2.8
	3	3.0	99.7	1.8	3.0	100.5	1.3	2.9	98.2	1.7	3.1	101.7	2.4
	40	42.3	105.7	1.2	41.1	102.9	1.2	41.2	103.1	2.5	41.5	103.8	1.0
Mesaconate	0.3	0.3	99.5	4.5	0.3	97.7	5.4	0.3	99.1	9.5	0.3	101.9	5.8
	3	3.0	101.1	1.0	3.1	102.0	1.2	3.0	101.5	1.8	3.2	105.1	2.2
	80	84.4	105.6	1.7	83.4	104.3	1.6	82.9	103.6	1.4	83.1	103.9	1.2
4-Octyl-itaconate	0.03	0.03	104.6	4.3	0.03	110.9	10.9	0.03	113.4	7.5	0.03	112.7	4.4
	0.3	0.3	88.0	3.0	0.3	89.1	2.8	0.3	90.3	2.0	0.3	99.8	1.0
	2	2.1	104.4	2.5	2.3	113.5	2.0	2.2	110.6	2.2	2.2	107.7	0.9
cis-Aconitate	0.15	0.1	95.6	14.0	0.1	96.9	5.3	0.1	97.3	3.6	0.1	94.5	3.4
	3	3.0	100.5	1.2	3.0	98.5	0.9	2.9	97.7	1.2	3.0	100.3	1.0
	40	42.9	107.3	0.6	41.8	104.6	1.7	41.8	104.6	0.5	42.3	105.8	1.4
Citrate	1.2	1.2	103.3	1.6	1.2	100.5	2.3	1.2	98.5	1.5	1.2	101.7	4.5
	6	6.3	105.7	0.9	6.1	101.5	0.8	6.1	101.1	1.8	6.2	102.7	1.4
	20	20.7	103.5	1.5	20.9	104.5	1.6	20.5	102.5	2.2	20.4	101.9	1.6
Fumarate	5	5.3	105.5	1.6	5.2	103.6	2.0	5.1	102.7	1.7	5.2	104.7	2.1
	12	12.9	107.5	1.2	12.4	103.0	0.7	12.4	103.0	2.2	12.9	107.3	1.9
	40	43.1	107.9	2.2	44.2	110.4	2.4	42.5	106.4	2.1	42.7	106.9	2.2
Isocitrate	0.6	0.6	99.7	0.8	0.6	99.6	3.0	0.6	96.5	4.8	0.6	96.4	5.8
	6	6.3	105.0	1.4	6.2	103.5	2.9	6.4	106.1	1.0	6.2	103.0	2.2
	40	41.1	102.7	2.7	40.0	100.0	2.1	39.1	97.7	2.8	41.2	103.0	3.0
Lactate	50	52.6	105.2	3.8	54.7	109.4	3.8	50.4	100.8	6.6	50.1	100.2	5.4
	150	158.2	105.5	2.3	159.0	106.0	6.0	158.4	105.6	0.8	157.0	104.7	1.7
	400	428.0	107.0	3.4	407.8	102.0	2.1	415.6	103.9	1.0	423.6	105.9	1.8
Malate	0.6	0.7	110.8	2.9	0.7	115.0	5.9	0.6	106.2	2.5	0.6	106.4	6.0
	6	6.6	109.5	1.5	6.6	109.2	1.7	6.6	110.7	0.4	6.3	105.2	1.4
	40	41.6	103.9	2.8	40.2	100.5	2.0	39.3	98.2	2.3	41.1	102.8	1.8
Succinate	5	4.3	85.8	1.8	4.3	86.1	5.1	4.1	82.8	2.0	4.2	83.0	2.2
	12	10.7	89.0	1.0	10.6	88.2	0.8	10.6	88.2	3.4	10.4	87.0	2.7
	40	38.1	95.4	2.4	39.1	97.8	2.0	37.8	94.5	2.5	37.3	93.3	2.5

Deviations >10% are underlined once; deviations >15%, which do not meet FDA recommendations, are underlined twice; *n* = 5 for each QC and time point.

Table S12. Long-term stability of quality controls in surrogate matrix at −20 °C.

QCs in Surrogate Matrix at −20 °C for	Exp. conc. (μM)	Mean (μM)	Accuracy (%)	Precision (%CV)	Mean (μM)	Accuracy (%)	Precision (%CV)	Mean (μM)	Accuracy (%)	Precision (%CV)	Mean (μM)	Accuracy (%)	Precision (%CV)	Mean (μM)	Accuracy (%)	Precision (%CV)
		1 week			1 month			2 months			6 months (ISTD ^{13}C Succinate)			6 months (ISTD ^{13}C Citrate)		
Itaconate	0.3	0.3	104.1	2.3	0.3	113.1	4.0	0.3	104.4	1.6	0.3	100.7	1.2			
	3	3.2	106.1	3.1	3.3	108.2	3.4	3.1	104.4	1.0	3.1	104.4	3.2			
	80	84.3	105.4	2.8	86.5	108.2	3.0	88.3	110.3	1.9	80.2	100.3	2.0			
Citraconate	0.15	0.2	104.4	4.5	0.2	101.9	7.5	0.2	106.7	4.7	0.2	97.9	2.5			
	3	3.1	104.5	2.3	3.2	105.5	3.3	3.1	103.7	1.0	3.2	107.2	2.1			
	40	42.4	106.0	3.3	43.6	109.0	3.0	44.2	110.6	2.2	39.7	99.4	2.1			
Mesaconate	0.3	0.3	102.7	4.8	0.3	110.0	2.7	0.3	110.7	2.7	0.3	95.9	1.9			
	3	3.2	105.7	2.7	3.2	107.5	4.1	3.2	105.5	0.7	3.2	106.3	2.8			
	80	85.3	106.7	3.1	89.1	111.4	3.7	88.5	110.7	2.6	80.2	100.3	1.6			
4-Octyl-itaconate	0.03	0.03	115.7	4.7	0.04	116.9	12.1	0.03	116.5	2.2	0.03	100.6	7.6			
	0.3	0.3	102.9	3.4	0.3	84.8	3.0	0.3	102.1	2.0	0.3	92.1	4.4			
	2	2.3	112.5	3.1	2.3	113.1	3.6	2.2	112.2	2.5	1.7	85.3	3.7			
cis-Aconitate	0.15	0.2	97.2	2.5	0.1	98.5	5.6	0.2	101.5	2.9	0.1	96.8	2.0			
	3	3.1	103.2	2.2	3.2	105.0	2.1	3.1	103.3	3.1	3.0	99.3	3.1			
	40	43.8	109.5	1.9	44.2	110.6	3.0	45.2	113.1	3.3	39.5	98.8	0.6			
Citrate	1.2	1.3	105.5	2.6	1.3	104.2	2.3	1.3	105.7	3.6	1.2	97.2	5.1			
	6	6.4	106.7	1.9	6.5	107.8	2.1	6.4	106.9	3.1	6.2	103.3	4.1			
	20	21.1	105.6	1.9	21.6	108.1	4.1	22.0	110.2	3.2	19.0	94.9	2.7			
Fumarate	5	5.6	111.7	2.0	5.6	111.1	1.3	5.5	110.7	1.9	4.9	97.0	1.6	5.1	101.2	3.2
	12	13.4	111.8	2.3	13.5	112.5	2.7	13.5	112.2	3.2	12.1	101.0	2.8	12.9	107.5	3.8
	40	45.5	113.9	3.2	45.2	113.1	5.3	45.9	114.8	3.2	39.8	99.6	2.8	37.0	92.6	4.0
Isocitrate	0.6	0.6	100.3	2.4	0.6	106.7	5.5	0.6	106.2	4.9	0.7	109.4	3.4			
	6	6.4	106.5	5.0	6.4	107.0	2.0	6.4	106.9	2.1	6.3	104.3	2.0			
	40	43.4	108.6	4.0	42.1	105.3	4.0	44.2	110.4	1.6	37.9	94.8	3.4			
Lactate	50	55.4	110.7	4.3	54.7	109.3	4.1	53.5	107.0	11.4	48.0	96.1	10.4			
	150	166.2	110.8	3.8	165.6	110.4	3.3	159.8	106.5	2.6	141.8	94.5	1.7			
	400	446.8	111.7	2.2	439.8	110.0	3.3	447.8	112.0	1.8	367.8	92.0	3.3			
Malate	0.6	0.7	108.3	4.1	0.7	112.5	5.4	0.7	118.3	8.9	0.6	103.0	7.4			
	6	6.6	109.8	5.0	6.6	109.5	2.3	6.6	110.5	2.7	6.4	106.9	2.7			
	40	42.8	106.9	3.0	43.0	107.4	2.5	43.7	109.3	2.2	37.7	94.2	2.8			
Succinate	5	4.4	87.3	2.6	4.2	84.5	2.4	4.9	97.3	2.6	4.6	92.2	4.0	4.8	96.4	5.2
	12	10.9	91.0	3.7	10.7	89.0	3.1	12.1	101.0	3.8	11.8	98.2	4.1	12.5	104.5	4.9
	40	39.2	98.0	2.7	39.7	99.4	6.2	42.7	106.7	2.4	38.2	95.4	3.2	33.3	83.2	4.6

Deviations >10% are underlined once; deviations >15%, which do not meet FDA recommendations, are underlined twice; *n* = 5 for each QC and time point.

Table S13. Base level concentrations of itaconate isomers and TCA intermediates in plasma/whole blood from donor C.

	Mean (µM)	Precision (%CV)	Mean (µM)	Precision (%CV)
	Plasma		Whole Blood	
Itaconate	<LLOQ		<LLOQ	
Citraconate	0.39	3.5	0.35	7.8
Mesaconate	<LLOQ		<LLOQ	
4-Octyl-itaconate	<LLOQ		<LLOQ	
cis-Aconitate	3.34	3.9	1.78	7.1
Citrate	123.24	0.9	63.45	4.4
Fumarate	<LLOQ		<LLOQ	
Isocitrate	40.06	2.6	19.86	6.9
Lactate	1247.20	2.0	898.50	7.1
Malate	10.58	1.4	23.20	7.1
Succinate	4.58	3.7	2.98	3.8

n = 5 aliquots from each biosample type.

Table S14. Stability of spiked-in itaconate isomers and derivatives in plasma from donor C at RT and 4 °C.

**Stability in Plasma**	**Exp. Conc. (µM)**	**Mean (µM)**	**Accuracy (%)**	**Precision (%CV)**	**Mean (µM)**	**Accuracy (%)**	**Precision (%CV)**	**Mean (µM)**	**Accuracy (%)**	**Precision (%CV)**	**Mean (µM)**	**Accuracy (%)**	**Precision (%CV)**
		0 h at RT			8 h at RT			24 h at RT			24 h at 4 °C		
Itaconate	0.3	0.3	108.5	2.9	0.3	107.9	2.0	0.3	106.4	2.2	0.3	107.1	1.8
	3	3.2	105.6	1.4	3.2	106.2	0.9	3.1	104.0	2.4	3.1	104.4	2.2
	80	82.6	103.3	0.9	81.7	102.1	1.0	82.0	102.6	2.8	81.8	102.3	1.7
Citraconate	0.15	0.1	84.7	16.2	0.0	-30.4	-22.2	0.0	-22.4	-37.2	0.0	4.3	94.4
	3	3.0	99.2	3.0	2.0	67.5	3.3	2.1	69.9	3.0	2.2	74.4	2.4
	40	40.4	101.1	1.3	39.6	99.0	1.2	40.2	100.6	2.2	39.9	99.8	2.0
Mesaconate	0.3	0.3	90.2	0.0	0.2	57.4	7.5	0.2	57.0	5.4	0.2	63.0	2.0
	3	2.7	89.7	0.1	1.9	62.5	2.8	1.9	63.1	1.7	2.0	65.8	1.6
	80	84.6	105.8	0.8	81.8	102.2	1.6	82.2	102.8	1.8	81.7	102.1	1.0
4-Octyl-itaconate	0.03	0.03	99.2	4.5	0.02	69.4	3.7	0.02	63.7	4.4	0.02	62.5	12.5
	0.3	0.3	94.6	6.5	0.2	71.1	2.5	0.2	63.3	4.2	0.2	63.5	7.1
	2	2.0	98.7	2.5	2.0	100.4	2.8	1.9	92.5	3.4	1.8	87.7	8.3

0 h samples were extracted 15 min after spike-in and aliquotation. Deviations >10% are underlined once; deviations >15%, which do not meet FDA recommendations, are underlined twice; n = 5 for each QC and time point.

Table S15. Stability of spiked-in itaconate isomers and derivatives in whole blood from donor C at 37 °C.

Stability in Whole Blood	Exp. Conc. (μM)	Mean (μM)	Accuracy (%)	Precision (%CV)	Mean (μM)	Accuracy (%)	Precision (%CV)	Mean (μM)	Accuracy (%)	Precision (%CV)
		0 h at 37 °C			4 h at 37 °C			8 h at 37 °C		
Itaconate	0.3	0.3	95.6	3.6	0.3	100.8	5.5	0.3	107.9	6.6
	3	2.8	92.7	2.2	2.9	95.8	2.4	3.1	103.3	6.1
	80	71.6	89.5	8.0	79.9	99.9	5.6	84.0	105.0	1.9
Citraconate	0.15	0.1	96.8	12.5	0.1	95.3	94.8	0.0	11.6	59.9
	3	2.8	93.9	2.8	2.5	84.7	1.3	2.4	80.3	8.2
	40	35.4	88.5	8.4	39.6	99.0	5.9	40.9	102.4	3.0
Mesaconate	0.3	0.2	65.6	2.6	0.2	60.9	8.7	0.2	55.6	4.7
	3	2.0	68.0	2.0	1.9	64.0	1.0	1.9	63.1	7.2
	80	69.5	86.8	8.4	78.6	98.2	6.2	82.1	102.6	2.1
4-Octyl-itaconate	0.03	0.02	66.5	4.0	0.02	61.2	12.6	0.02	51.9	5.0
	0.3	0.2	68.6	4.8	0.2	63.1	2.7	0.2	57.8	9.1
	2	1.7	85.3	8.7	1.8	92.0	4.8	1.9	94.5	2.3

0 h samples were extracted 15 min after spike-in and aliquotation. Deviations >10% are underlined once; deviations >15%, which do not meet FDA recommendations, are underlined twice; *n* = 5 for each QC and time point.

Table S16. Stability of spiked-in TCA metabolites in plasma from donor C at RT and 4 °C.

Stability in Plasma	Exp. Conc. (µM)	Mean (µM)	Accuracy (%)	Precision (%CV)	Mean (µM)	Accuracy (%)	Precision (%CV)	Mean (µM)	Accuracy (%)	Precision (%CV)	Mean (µM)	Accuracy (%)	Precision (%CV)
		0 h at RT			8 h at RT			24 h at RT			24 h at 4 °C		
cis-Aconitate	0.15	0.3	214.7	42.4	0.7	444.0	11.7	0.7	462.7	11.8	0.1	70.7	153.5
	3	3.0	99.0	5.3	2.2	71.7	7.7	2.0	65.5	1.1	1.7	57.4	16.8
	40	37.3	93.3	1.8	20.0	50.0	3.0	16.6	41.4	1.2	19.2	48.0	6.4
Citrate	1.2	-4.5	-376.7	52.0	0.9	73.3	152.5	-0.6	-46.7	436.0	7.9	656.7	56.1
	6	1.8	30.0	148.6	7.1	118.7	101.6	0.5	8.0	384.2	23.0	384.0	28.1
	20	10.2	50.8	57.9	22.9	114.6	19.1	25.2	126.2	16.6	40.6	203.2	13.5
Fumarate	5	4.3	85.5	9.1	2.6	52.2	11.6	1.5	29.6	29.0	2.4	47.7	12.6
	12	12.0	100.3	5.2	8.1	67.5	4.5	4.7	39.6	7.0	7.8	65.0	10.7
	40	41.3	103.2	1.2	30.3	75.8	4.7	21.8	54.6	2.5	29.8	74.6	5.2
Isocitrate	0.6	2.7	456.7	44.7	6.5	1090.0	16.3	8.3	1383.3	12.0	13.4	2236.7	13.2
	6	26.5	441.0	5.2	29.7	495.0	11.9	30.9	515.7	3.9	44.6	743.7	7.1
	40	155.5	388.8	4.1	164.4	411.0	3.1	169.1	422.9	2.5	206.3	515.9	6.1
Lactate	50	57.2	114.4	62.5	185.2	370.4	21.4	244.4	488.8	18.4	285.2	570.4	29.1
	150	144.8	96.5	30.9	275.2	183.5	17.6	389.2	259.5	14.5	504.4	336.3	19.3
	400	352.4	88.1	22.3	483.6	120.9	5.8	548.0	137.0	8.7	703.2	175.8	18.0
Malate	0.6	-1.0	-162.7	43.5	1.6	260.7	30.5	5.5	911.3	8.5	3.8	638.7	15.3
	6	8.2	136.7	10.1	13.4	223.0	8.8	19.3	321.0	2.1	19.5	324.3	11.5
	40	59.7	149.3	3.4	70.8	177.1	2.3	85.1	212.9	2.1	87.6	219.1	6.6
Succinate	5	3.1	61.7	9.1	2.9	57.3	13.1	3.3	66.6	3.3	2.7	54.2	7.6
	12	7.0	58.7	3.9	6.8	56.5	3.4	7.3	60.9	5.8	6.7	56.0	4.5
	40	23.1	57.9	3.3	22.4	56.0	4.3	22.8	57.0	3.7	23.1	57.7	1.1

0 h samples were extracted 15 min after spike-in and aliquotation. Deviations > 10% are underlined once; deviations > 15%, which do not meet FDA recommendations, are underlined twice; *n* = 5 for each QC and time point.

Table S17. Stability of spiked-in TCA metabolites in whole blood from donor C at 37 °C

Stability in Whole Blood	Exp. Conc. (µM)	Mean (µM)	Accuracy (%)	Precision (%CV)	Mean (µM)	Accuracy (%)	Precision (%CV)	Mean (µM)	Accuracy (%)	Precision (%CV)
		0 h at 37 °C			4 h at 37 °C			8 h at 37 °C		
cis-Aconitate	0.15	0.2	157.3	34.6	1.3	868.0	11.9	1.4	921.3	12.6
	3	2.2	74.5	5.8	2.4	78.6	2.9	2.5	83.9	8.8
	40	27.8	69.4	6.7	18.6	46.5	4.4	16.4	40.9	2.3
Citrate	1.2	-3.4	-280.8	51.7	-5.9	-490.8	33.8	-7.4	-617.5	11.2
	6	-2.1	-35.5	103.6	-3.1	-50.8	37.8	-1.6	-26.2	159.2
	20	5.7	28.8	103.2	15.8	79.2	20.4	18.4	92.0	6.5
Fumarate	5	4.9	98.5	20.4	6.9	137.9	5.0	8.7	173.1	9.9
	12	11.2	93.4	6.7	9.1	76.0	3.5	11.3	94.3	9.2
	40	34.0	85.1	10.5	21.9	54.7	7.5	23.7	59.2	6.9
Isocitrate	0.6	5.1	843.3	31.9	16.6	2763.3	9.6	19.1	3183.3	4.9
	6	29.3	487.7	8.7	45.0	750.3	1.8	52.1	867.7	8.3
	40	134.8	337.0	10.1	200.5	501.4	5.4	234.9	587.4	1.7
Lactate	50	573.6	1147.2	5.8	5313.2	10626.4	3.9	9025.2	18050.4	3.0
	150	637.6	425.1	10.1	5285.2	3523.5	2.5	9165.2	6110.1	6.0
	400	825.2	206.3	13.3	5697.2	1424.3	5.5	9541.2	2385.3	1.4
Malate	0.6	3.9	646.7	27.7	58.0	9660.0	3.3	99.2	16526.7	5.3
	6	11.7	195.3	4.1	69.8	1163.3	2.8	112.0	1866.7	7.2
	40	64.4	161.0	7.8	154.8	387.0	5.1	196.4	491.0	1.3
Succinate	5	1.8	36.6	9.0	2.8	55.4	11.6	4.0	80.4	9.7
	12	5.0	41.9	7.5	5.8	48.0	3.8	7.3	60.8	7.3
	40	16.8	42.1	9.6	20.7	51.8	6.3	22.9	57.3	2.2

0 h samples were extracted 15 min after spike-in and aliquotation. Deviations > 10% are underlined once; deviations > 15%, which do not meet FDA recommendations, are underlined twice; *n* = 5 for each QC and time point.

Table S18. Accuracy and precision of QC-H dilutions.

	Exp. Conc. (µM)	Mean (µM)	Accuracy (%)	Precision (%CV)
	QC-H 1:10	Diluted Extracted Samples		
Itaconate	8	8.5	106.7	1.9
Citraconate	4	4.2	105.8	1.6
Mesaconate	8	8.7	108.9	1.9
4-Octyl-itaconate	0.2	0.2	109.0	2.1
cis-Aconitate	4	4.3	106.2	1.7
Citrate	2	1.9	93.3	2.9
Fumarate	4	4.4	110.2	2.6
Isocitrate	4	4.8	120.9	3.6
Lactate	40	49.6	124.0	5.7
Malate	4	5.7	142.3	2.3
Succinate	4	4.2	106.1	1.8

Deviations > 10% are underlined once; deviations > 15%, which do not meet FDA recommendations, are underlined twice; *n* = 5.

References:

12. Nguyen, T.V.; Alfaro, A.C.; Merien, F.; Young, T.; Grandiosa, R. Metabolic and immunological responses of male and female new Zealand Greenshell mussels (Perna canaliculus) infected with Vibrio sp. *J. Invertebr. Pathol.* **2018**, *157*, 80–89, doi:10.1016/j.jip.2018.08.008.
13. Aggrey, S.E.; Milfort, M.C.; Fuller, A.L.; Yuan, J.; Rekaya, R. Effect of host genotype and Eimeria acervulina infection on the metabolome of meat-type chickens. *PLoS One* **2019**, *14*, e0223417, doi:10.1371/journal.pone.0223417.
14. Michopoulos, F.; Karagianni, N.; Whalley, N.M.; Firth, M.A.; Nikolaou, C.; Wilson, I.D.; Critchlow, S.E.; Kollias, G.; Theodoridis, G.A. Targeted Metabolic Profiling of the Tg197 Mouse Model Reveals Itaconic Acid as a Marker of Rheumatoid Arthritis. *J. Proteome Res.* **2016**, *15*, 4579–4590, doi:10.1021/acs.jproteome.6b00654.
15. Li, Y.; Liang, L.; Deng, X.; Zhong, L. Lipidomic and metabolomic profiling reveals novel candidate biomarkers in active systemic lupus erythematosus. *Int. J. Clin. Exp. Pathol.* **2019**, *12*, 857–866.
16. Mossa, A.H.; Shamout, S.; Cammisotto, P.; Campeau, L. Urinary metabolomics predict the severity of overactive bladder syndrome in an aging female population. *Int. Urogynecol. J.* **2019**, 10.1007/s00192-019-04175-6, doi:10.1007/s00192-019-04175-6.
17. Meiser, J.; Kraemer, L.; Jaeger, C.; Madry, H.; Link, A.; Lepper, P.M.; Hiller, K.; Schneider, J.G. Itaconic acid indicates cellular but not systemic immune system activation. *Oncotarget* **2018**, *9*, 32098–32107, doi:10.18632/oncotarget.25956.
18. Duran, M.; Bruinvis, L.; Ketting, D.; Wadman, S.K. Deranged isoleucine metabolism during ketotic attacks in patients with methylmalonic acidaemia. *J. Inherit. Metab. Dis.* **1978**, *1*, 105–107, doi:10.1007/bf01805683.
19. Zhu, J.; Djukovic, D.; Deng, L.; Gu, H.; Himmati, F.; Abu Zaid, M.; Chiorean, E.G.; Raftery, D. Targeted serum metabolite profiling and sequential metabolite ratio analysis for colorectal cancer progression monitoring. *Anal. Bioanal. Chem.* **2015**, *407*, 7857–7863, doi:10.1007/s00216-015-8984-8.
20. Wang, H.; Zhang, H.; Yao, L.; Cui, L.; Zhang, L.; Gao, B.; Liu, W.; Wu, D.; Chen, M.; Li, X., et al. Serum metabolic profiling of type 2 diabetes mellitus in Chinese adults using an untargeted GC/TOFMS. *Clin. Chim. Acta* **2018**, *477*, 39–47, doi:10.1016/j.cca.2017.11.036.
21. Eguchi, A.; Sakurai, K.; Watanabe, M.; Mori, C. Exploration of potential biomarkers and related biological pathways for PCB exposure in maternal and cord serum: A pilot birth cohort study in Chiba, Japan. *Environ. Int.* **2017**, *102*, 157–164, doi:10.1016/j.envint.2017.02.011.
22. Hao, J.; Yang, T.; Zhou, Y.; Gao, G.Y.; Xing, F.; Peng, Y.; Tao, Y.Y.; Liu, C.H. Serum Metabolomics Analysis Reveals a Distinct Metabolic Profile of Patients with Primary Biliary Cholangitis. *Sci. Rep.* **2017**, *7*, 784, doi:10.1038/s41598-017-00944-9.
23. De Biase, I.; Pasquali, M.; Asamoah, A. Unusual Metabolites in a Patient with Isovaleric Acidemia. *Clin. Chem.* **2019**, *65*, 595–597, doi:10.1373/clinchem.2018.300558.
24. Coman, D.; Vissers, L.; Riley, L.G.; Kwint, M.P.; Hauck, R.; Koster, J.; Geuer, S.; Hopkins, S.; Hallinan, B.; Sweetman, L., et al. Squalene Synthase Deficiency: Clinical, Biochemical, and Molecular Characterization of a Defect in Cholesterol Biosynthesis. *Am. J. Hum. Genet.* **2018**, *103*, 125–130, doi:10.1016/j.ajhg.2018.05.004.

28. Shin, J.H.; Yang, J.Y.; Jeon, B.Y.; Yoon, Y.J.; Cho, S.N.; Kang, Y.H.; Ryu, D.H.; Hwang, G.S. (1)H NMR-based metabolomic profiling in mice infected with Mycobacterium tuberculosis. *J. Proteome Res.* **2011**, *10*, 2238–2247, doi:10.1021/pr101054m.

29. Zhu, X.; Lei, H.; Wu, J.; Li, J.V.; Tang, H.; Wang, Y. Systemic responses of BALB/c mice to Salmonella typhimurium infection. *J. Proteome Res.* **2014**, *13*, 4436–4445, doi:10.1021/pr500770x.

52. Beloborodova, N.; Pautova, A.; Sergeev, A.; Fedotcheva, N. Serum Levels of Mitochondrial and Microbial Metabolites Reflect Mitochondrial Dysfunction in Different Stages of Sepsis. *Metabolites* **2019**, *9*, doi:10.3390/metabo9100196.
53. de Seymour, J.V.; Conlon, C.A.; Sulek, K.; Villas Boas, S.G.; McCowan, L.M.; Kenny, L.C.; Baker, P.N. Early pregnancy metabolite profiling discovers a potential biomarker for the subsequent development of gestational diabetes mellitus. *Acta Diabetol* **2014**, *51*, 887–890, doi:10.1007/s00592-014-0626-7.
54. Riquelme, S.A.; Liimatta, K.; Wong Fok Lung, T.; Fields, B.; Ahn, D.; Chen, D.; Lozano, C.; Saenz, Y.; Uhlemann, A.C.; Kahl, B.C., et al. Pseudomonas aeruginosa Utilizes Host-Derived Itaconate to Redirect Its Metabolism to Promote Biofilm Formation. *Cell Metab.* **2020**, *31*, 1091–1106 e1096, doi:10.1016/j.cmet.2020.04.017.
55. Van Nguyen, T.; Alfaro, A.C. Targeted metabolomics to investigate antimicrobial activity of itaconic acid in marine molluscs. *Metabolomics* **2019**, *15*, 97, doi:10.1007/s11306-019-1556-8.
56. Shao, C.; Song, J.; Zhao, S.; Jiang, H.; Wang, B.; Chi, A. Therapeutic Effect and Metabolic Mechanism of A Selenium-Polysaccharide from Ziyang Green Tea on Chronic Fatigue Syndrome. *Polymers* **2018**, *10*, doi:10.3390/polym10111269.
57. Weiss, J.M.; Davies, L.C.; Karwan, M.; Ileva, L.; Ozaki, M.K.; Cheng, R.Y.; Ridnour, L.A.; Annunziata, C.M.; Wink, D.A.; McVicar, D.W. Itaconic acid mediates crosstalk between macrophage metabolism and peritoneal tumors. *J. Clin. Invest.* **2018**, *128*, 3794–3805, doi:10.1172/JCI99169.
58. Shi, R.; Zhang, J.; Fang, B.; Tian, X.; Feng, Y.; Cheng, Z.; Fu, Z.; Zhang, J.; Wu, J. Runners' metabolomic changes following marathon. *Nutr. Metab.* **2020**, *17*, 19, doi:10.1186/s12986-020-00436-0.
59. Kalantari, S.; Nafar, M.; Samavat, S.; Parvin, M. 1h NMR-based metabolomics study for identifying urinary biomarkers and perturbed metabolic pathways associated with severity of IgA nephropathy: a pilot study. *Magn. Reson. Chem.* **2017**, *55*, 693–699, doi:10.1002/mrc.4573.
60. Q Wu; X Lai;h Zhao; Z Zhu; Z Hong; Z Guo; Chai, Y. A metabolomics approach for predicting the response to intravenous iron therapy in peritoneal dialysis patients with anemia. *RSC Adv.* **2017**, *7*, 1915–1922.
61. Tasic, L.; Pontes, J.G.M.; Carvalho, M.S.; Cruz, G.; Dal Mas, C.; Sethi, S.; Pedrini, M.; Rizzo, L.B.; Zeni-Graiff, M.; Asevedo, E., et al. Metabolomics and lipidomics analyses by (1)H nuclear magnetic resonance of schizophrenia patient serum reveal potential peripheral biomarkers for diagnosis. *Schizophr. Res.* **2017**, *185*, 182–189, doi:10.1016/j.schres.2016.12.024.
62. Maeda, E.; Matsuo, M.; Saiki, K.; Nakamura, H.; Matsuo, T.; Takemine, H. Methylsuccinate and mesaconate in urine of patients treated with sodium benzoate. *Acta Paediatr. Jpn.* **1989**, *31*, 721–726, doi:10.1111/j.1442-200x.1989.tb01386.x.
63. Shigematsu, Y.; Sudo, M.; Momoi, T.; Inoue, Y.; Suzuki, Y.; Kameyama, J. Changing plasma and urinary organic acid levels in a patient with isovaleric acidemia during an attack. *Pediatr. Res.* **1982**, *16*, 771–775, doi:10.1203/00006450-198209000-00013.
64. Truscott, R.J.; Malegan, D.; McCairns, E.; Burke, D.; Hick, L.; Sims, P.; Halpern, B.; Tanaka, K.; Sweetman, L.; Nyhan, W.L., et al. New metabolites in isovaleric acidemia. *Clin. Chim. Acta* **1981**, *110*, 187–203, doi:10.1016/0009-8981(81)90348-x.
65. Liebich, H.M.; Pickert, A.; Stierle, U.; Woll, J. Gas chromatography-mass spectrometry of saturated and unsaturated dicarboxylic acids in urine. *J. Chromatogr.* **1980**, *199*, 181–189, doi:10.1016/s0021-9673(01)91371-8.
66. Nakamura, E.; Rosenberg, L.E.; Tanaka, K. Microdetermination of methylmalonic acid and other short chain dicarboxylic acids by gas chromatography: use in prenatal diagnosis of methylmalonic acidemia and in studies of isovaleric acidemia. *Clin. Chim. Acta* **1976**, *68*, 127–140, doi:10.1016/0009-8981(76)90412-5.
67. Gao, L.; Yuan, H.; Xu, E.; Liu, J. Toxicology of paraquat and pharmacology of the protective effect of 5-hydroxy-1-methylhydantoin on lung injury caused by paraquat based on metabolomics. *Sci. Rep.* **2020**, *10*, 1790, doi:10.1038/s41598-020-58599-y.
68. Montgomery, J.A.; Mamer, O.A.; Scriver, C.R. Metabolism of ethylmalonate to mesaconate in the rat. Evidence for trans-dehydrogenation of methylsuccinate. *Biochem. J.* **1983**, *214*, 641–644, doi:10.1042/bj2140641.
69. Su, W.; Shi, J.; Zhao, Y.; Yan, F.; Lei, L.; Li, H. Porphyromonas gingivalis triggers inflammatory responses in periodontal ligament cells by succinate-succinate dehydrogenase-HIF-1alpha axis. *Biochem. Biophys. Res. Commun.* **2020**, *522*, 184–190, doi:10.1016/j.bbrc.2019.11.074.
70. Shigematsu, M.; Tomonaga, S.; Shimokawa, F.; Murakami, M.; Imamura, T.; Matsui, T.; Funaba, M. Regulatory responses of hepatocytes, macrophages and vascular endothelial cells to magnesium deficiency. *J. Nutr. Biochem.* **2018**, *56*, 35–47, doi:10.1016/j.jnutbio.2018.01.008.
